# Mapping the Chiroptical Properties of Local Domains in Thin Films of Chiral Silicon Phthalocyanines by CD Imaging

**DOI:** 10.3390/molecules25246048

**Published:** 2020-12-21

**Authors:** Dora-M. Răsădean, Tiberiu-M. Gianga, Tamás Jávorfi, Rohanah Hussain, Giuliano Siligardi, G. Dan Pantoș

**Affiliations:** 1 Department of Chemistry, University of Bath, Claverton Down, Bath BA2 7AY, UK; dmr35@bath.ac.uk (D.-M.R.); tiberiu-marius.gianga@diamond.ac.uk (T.-M.G.); 2 Beamline B23, Diamond Light Source, Ltd., Chilton, Didcot OX11 0DE, UK; tamas.javorfi@diamond.ac.uk (T.J.); rohanah.hussain@diamond.ac.uk (R.H.); giuliano.siligardi@diamond.ac.uk (G.S.)

**Keywords:** chiral thin films, phthalocyanines, synchrotron radiation, circular dichroism, imaging

## Abstract

The first example of uniformly chiral thin films of silicon phthalocyanines (SiPcs) are reported. The local domains of the films are mapped using circular dichroism (CD) imaging (CD*i*) technique available at the Diamond B23 beamline. The CD*i* allowed us to increase the spatial resolution up to 525× when compared with benchtop spectrometers. The results indicate formation on-surface of chiral and stable supramolecular assemblies with homogenous distribution. Chemical functionalization and solvent choice for deposition allow controllable chiroptical properties to be obtained. The method and technique reported in this work could be applied to prepare and characterize a wide variety of chiral thin films.

## 1. Introduction

Organic materials have emerged as promising candidates for optoelectronic applications based on their flexibility and low cost of production [1]. Among these, metallophthalocyanines (Pcs) are of particular interest due to their high thermal and chemical stabilities [2,3,4,5]. Pcs are semiconductors [2,3,6,7] with excellent optical and electrical properties, being widely used for write-once read-many materials, organic photovoltaics (OPVs), organic light emitting diodes (OLEDs), organic thin-film transistors (OTFTs), non-linear optical devices, etc. [2,4,6,7,8]. Representative examples for such applications include thin films of Cu, Zn, Co, Pb, Ni, Sn, TiO, VO Pcs deposited on a variety of substrates [1]. The performance of Pcs-based devices depends on the solid-state arrangement [4] of molecules, which is in part dependent on the deposition method. Numerous deposition methods have been developed for this, including vacuum thermal deposition, Langmuir–Blodgett, spin-coating and drop casting [1,6]. Another way to improving the films and devices properties is through chemical functionalization of the Pc core. Pcs coordinated onto divalent metals can only be functionalized at the periphery, which imposes some limitations [4]. Pcs with tetravalent metals provide additional flexibility through axial functionalization, with SiPcs recently finding applications in OPVs and OLEDs fabrication [4,9]. Chirality can be used to control the self-assembly of molecules on surfaces, however, there are only three reports of chiral Pcs as precursors for thin films [10,11,12]. Here we present a new way of producing and characterizing chiral Pc-based thin films.

Alongside the advances in producing chiral thin films, significant progress has been made towards specific analysis methods. These include microscopy techniques and electronic circular dichroism (ECD) spectroscopy [13,14]. Microscopy reveals film morphology while ECD allows the study of chirality of π-conjugated systems [13,15]. Electronic circular dichroism imaging (CD*i*) has recently emerged as an innovative method to complement these techniques [15,16,17]. CD*i*, which we use to characterize our thin films, is a powerful tool developed at the Diamond Light Source B23 (DLS B23) beamline for synchrotron radiation circular dichroism (SRCD). CD*i* enables mapping the chirality of local domains in thin films at spatial resolutions as low as 0.05 mm [15,16]. This is made possible by the unique, highly collimated beam of DLS B23, and cannot be achieved with benchtop ECD spectropolarimeters.

## 2. Results and Discussion

Herein, we report the first examples of chiral thin films obtained by on surface supramolecular assembly of chiral SiPc and their characterization via CD*i* and 3D surface profilometry.

**Figure 1:**
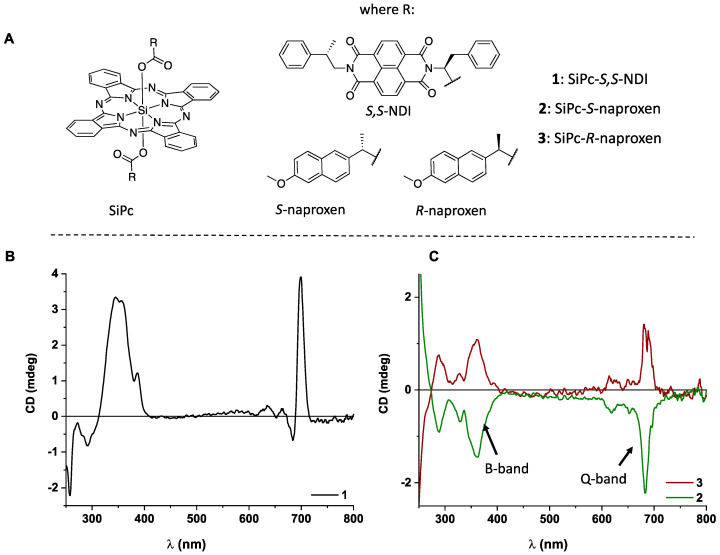
(**A**) Chemical structures of metallophthalocyanines (Pcs) used in this work; (**B**) electronic circular dichroism (ECD) spectrum of **1** (black line); (**C**) ECD spectra of **2** (green line) and **3** (brown line) solutions in CH_2_Cl_2_ (2 × 10^−5^ M). The main spectral features B- and Q-bands of Pcs are shown in C [19].

The SiPcs used in this study are shown in Figure 1A. We have recently reported a one-step, microwave-assisted approach to synthesize chiral SiPcs through axial coordination [18]. The three SiPcs used in this study have been synthesized via this method (full characterization in the Materials and Methods section). The three SiPcs share common features of axial ligands bearing aromatic surfaces and chiral centers close to the Pc ring. They were chosen due to the large Cotton effect observed on both the B and Q-phthalocyanine bands [18].

Molecule **1** is a naphthalenediimide (NDI) derivative, while **2** and **3** are a pair of enantiomers based on naproxen coordinated on the SiPc core. ECD studies show that, in solution, the chiral information is transferred from the ligand onto the achiral SiPc core (Figure 1B,C). An ECD response is observed for both Q-band (around 690 nm) and B-band (below 400 nm), which are spectral signatures of Pcs [19] (Figure 1C). The SiPcs **1**–**3** are soluble in common organic solvents due to their axial substituents and are suitable for most deposition techniques. We have chosen the drop-cast method due to its advantages of simple operation, minimal material loss and low cost [1,5]. We have used fused silica as substrate for film deposition as it is transparent in the far-ultraviolet (UV) region and provides a robust support resistant to temperature variations. We have chosen solvents of different polarities, with high boiling points and good adhesion properties in which SiPcs are soluble: *N*,*N*-dimethylformamide (DMF) and 1,1,2,2-tetrachloroethane (TCE). Both DMF and TCE have high enough boiling points to prevent fast evaporation of solvent and excessive drying pattern formation. Adhesivity to the substrate is directly linked to surface tension and viscosity of the solvent; the higher these variables, the better the solution adheres to fused silica. DMF and TCE have higher surface tensions (72 and 36 mN × m^−1^, respectively) than other solvents in which Pcs are usually soluble (e.g., CH_2_Cl_2_ and toluene: 27 and 30 mN × m^−1^, respectively). Viscosity follows a similar trend, with DMF and TCE being more viscous than CH_2_Cl_2_ and toluene: 0.764 and 1.437 versus 0.413 and 0.560 mPa × s, in that order [20]. Thin films were prepared by drop-casting 40 µL from stock solutions of 10^−3^ M in DMF (**1**) and TCE (**1**–**3**) on fused silica plates, followed by evaporation of solvent (detailed procedure in Materials and Methods section and Figures S4 and S8). The on-surface supramolecular assemblies are chiral due to the point-chirality present in Pcs. Solution studies (Figure 1B,C) have shown that the chiral information is visible in both the Q- and B-bands. The films were analyzed in the B-band region due to better signal-to-noise ratio of the DLS B23 CD*i* setup at these wavelengths.

One of the most accurate representations of the ECD response in thin films is the dissymmetry *g*-factor. The *g*-factor is calculated as shown in Equation (1):

(1) $$g=\;\frac{2(I_{L\;}-I_{R})}{I_{L}+\;I_{R}}$$

where *I_L_* and *I_R_* are the absorption intensities of left- and right-handed circularly polarized light [21]. This is a dimensionless measurement unit that eliminates any contributions of a film’s thickness to the ECD signal. The molar circular dichroism, necessary for the calculation of the *g*-factor, was calculated by dividing the molar ellipticity measured in mdeg by 32,980 [22]. The *g*-factor ranges between −2 and +2, spanning between 10^−3^ and 10^−4^ for organic molecules [21].

**Figure 2:**
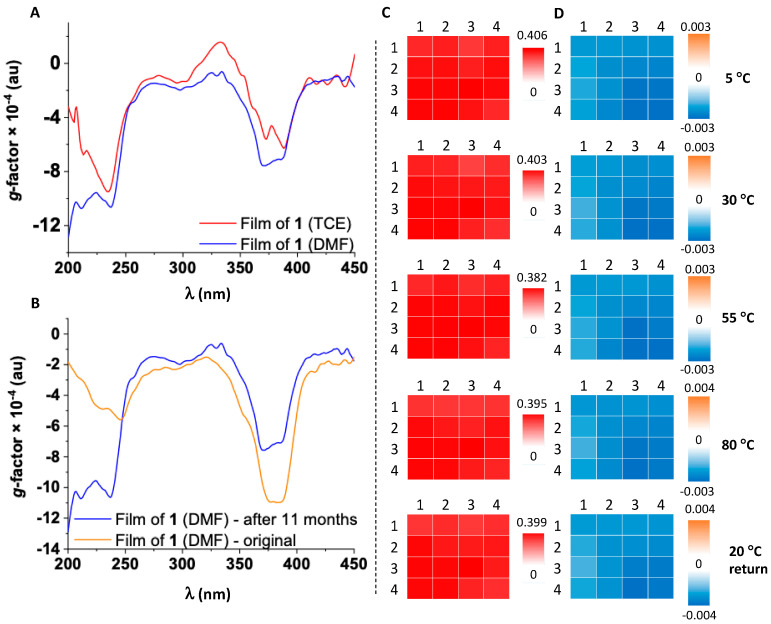
(**A**) Overlaid local dissymmetry *g*-factor spectra of thin films of **1** from 1,1,2,2-tetrachloroethane (TCE, red line) and *N*,*N*-dimethylformamide (DMF, blue line); (**B**) Overlaid local dissymmetry *g*-factor spectra of thin film of **1** freshly made from DMF (orange line) and the same film after kept 11 months at room temperature (blue line); (**C**) variable temperature (VT) ultraviolet–visible (UV–vis) absorption 2D maps of thin film of **1**; (**D**) VT dissymmetry *g*-factor 2D maps of thin film of **1** at the specified temperatures. The data was generated by circular dichroism imaging (CD*i*): 4 × 4 grid array area of 1 mm step size with a beam diameter of about 0.05 mm at 387 nm.

Derivative **1** has flexible sidechains appended to the aromatic NDI, displaying the largest ellipticity in solution as shown by our previous studies [18]. The absorption pattern of film of **1** from TCE is similar to that in solution. The ECD spectrum shows a bisignate profile with a magnitude of ~20 mdeg in the B-band and *g*-factor of 10^−4^ (Figure 2A). CD*i* 2D maps of local areas (of 25 mm^2^) of this film are homogeneous in ultraviolet–visible (UV–vis), ECD, and *g*-factor domains (Figures S30–S36).

**Figure 3:**
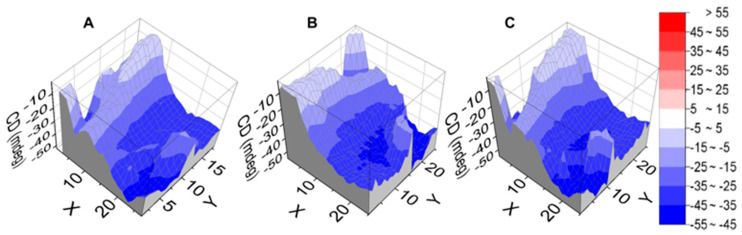
Three-dimensional ECD maps of film of **1** from DMF at 391 nm: (**A**) top; (**B**) bottom; (**C**) bottom flipped along the *y*-axis, indicating good degree of homogeneity and lack of linear dichroism and linear birefringence (LDLB) contributions; the steps for *x*- and *y*-axes are represented every 0.2 mm.

The influence of drop casting solvent on the properties of thin films was tested by dissolving **1** in DMF, followed by film preparation as described above. The absorption spectrum has broader bands than the film produced from TCE, suggesting the formation of a less ordered film. The ECD profile (represented as dissymmetry *g*-factor spectra in Figure 2A) shows two main negative bands at around 390 and 250 nm, similar to the films made from TCE. CD*i* maps of the B-band (overlapped with NDI absorption at 391 nm) and the 237 nm regions show homogeneous coverage across local domains of 25 mm^2^ (Figures S12–S17). The *g*-factor of a square 5 × 5 m^2^ area of **1** for top and bottom orientations is less than 10^−2^, which indicates that both magnetic (m) and electric (µ) dipole transition moments are forbidden. Both top and bottom 3D maps, flipped along the *y*-axis, of the *g*-factor retained the same sign (Figure S44). The top (Figure 3A) and bottom flipped (Figure 3C) maps for this sample are very similar and are consistent with the absence of polarization effects.

The stability of films is of paramount importance for their incorporation into devices. We have analyzed the stability of a film of **1** by ECD spectroscopy using CD*i* variable temperature (VT) studies available at DLS B23 beamline. We have mapped local domains of 4 × 4 mm^2^ across 300–450 nm (Figure 2C,D), acquiring 16 spectra at each temperature over a range of 5–80 °C every 25 °C. Both absorption and dissymmetry *g*-factor maps display a high degree of similarity with negligible changes across the temperature range (Figure 2C,D). This indicates that the supramolecular assembly formed on the surface is stable between 5–80 °C. The chiroptical response maintains its shape, but increases in magnitude with almost 3 mdeg (~10^−4^ *g*-factor units) over temperature ramp, returning to initial state when the film was cooled back to 20 °C (ECD maps shown in Figure S30).

The robustness of the supramolecular structure has been also tested by recording the absorption and ECD spectra of an 11 month-old film kept at room temperature (Figures S5 and S9). The spectra are similar to those of a freshly made film (Figure 2B), confirming that NDI-based SiPc arranges into highly robust films containing chiral aggregates on fused silica surface independent of the solvent/moisture content.

**Figure 4:**
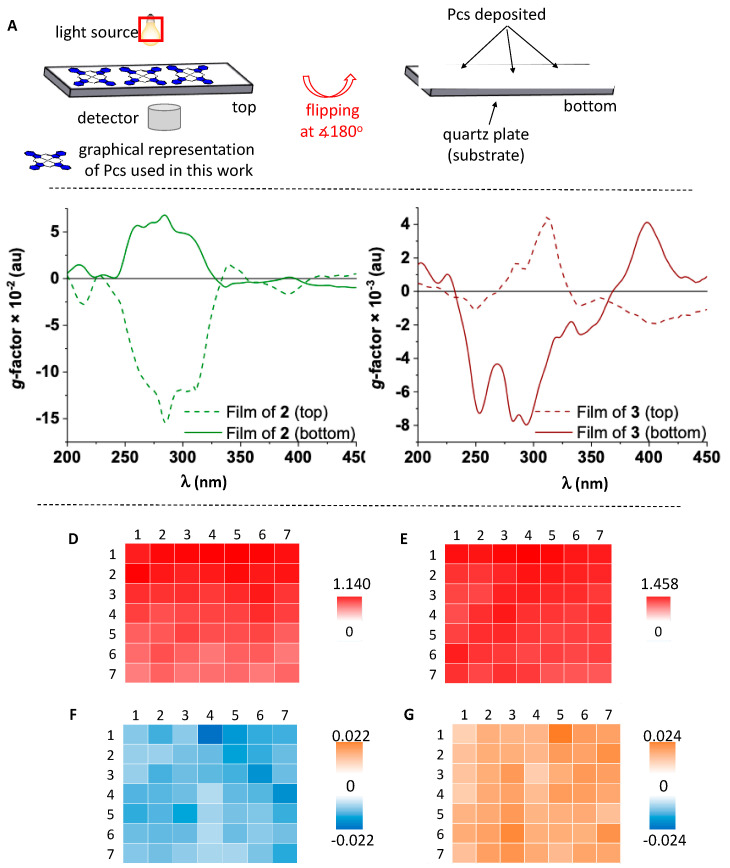
(**A**) Schematic representation of fused silica plate flipping to allow top and bottom analysis of films of **1**–**3**; (**B**,**C**) Local dissymmetry *g*-factor spectra of thin films of **2** and **3** from TCE, respectively, top (dashed line) and bottom (solid line); (**D**,**E**) UV–vis absorption 2D maps of top (**D**) and bottom (**E**) sides of film of **3**; (**F**,**G**) dissymmetry *g*-factor 2D maps of top (**F**) and bottom (**G**) sides of film of **3**. The data was generated by CD*i*: 7 × 7 grid array area of 0.2 mm step size with a beam diameter of 0.05 mm at 391 nm.

The naproxen ligand of the enantiomeric pair **2** and **3** is less flexible than the NDI and allows us to study the influence of the axial ligand on the film morphology and chiroptical properties. Only TCE was used as solvent for these films due to its higher viscosity compared to DMF, which facilitates thin film production. The UV–vis absorption spectra of films of **2** and **3** show broad bands with a similar pattern to the UV–vis spectra of these molecules in solution. The B-band of Pc core has a maximum at around 350 nm, while the naproxen unit absorbs across 250–310 nm (Figures S10 and S11). The corresponding ECD spectra display bisignate Cotton effects with a maximum intensity at 285 nm for **2** and 312 nm for **3** (Figures S6 and S7 in mdeg and *g*-factor spectra in Figure 3B,C). The mismatch in wavelength maxima (i.e., spectra not perfect mirror image) arises from defects at a macroscopic level and scattering phenomena, the latter being a common issue in thin films. The local magnitude of the two films is different because of the unequal amount of Pc deposited on that particular area. Both films of molecules **2** and **3** have local domains with large ellipticities. These are produced by the structural alignment of molecules on the surface, leading to linear anisotropy. We have used the “top and bottom” method, in which the fused silica plate is flipped at 180°, in order to demonstrate these contributions (Figure 4A).

The actual ECD response is not affected by rotation or flipping of the films. Thus, its sign should not change upon sample flipping if linear effects make negligible contributions as in the case of molecule **1** (maps of top side of film of **1** are shown in Figure 2C,D and bottom side maps are given in Figures S18–S29). However, the ECD spectra of films of **2** and **3** show an opposite sign upon sample flipping (Figure 4B,C, solid lines). This is due to the coupling of linear dichroism (LD) and linear birefringence (LB) contributions, which are polarization effects that arise from sample orientation and are intrinsic anisotropic components of ECD, usually being referred to as the LDLB effect [16,18,23]. The dissymmetry *g*-factor of naproxen-functionalized SiPcs has values as large as 10^−1^ (film of **2** in Figure 4B) and 10^−2^ (film of **3** in Figure 4C). These magnitudes of the *g*-factor are due to the LDLB effect and are unprecedented for phthalocyanine films, being essential for obtaining sizeable dichroic effects [24] required for the development of chiroptical sensors. These large *g*-factors exceed those of other organic molecules films previously reported such as π-conjugated polymers [16,18,23].

Mapping was carried out for the B-band of the Pc core (391 nm) as well as the ligand (311 nm). The absorption maps of **3** (Figure 4D,E) and **2** (Figures S37–S42) show a high degree of homogeneity for both regions, suggesting similar distributions of chromophores across the mapped area and supramolecular chirality. The LDLB contribution was assessed by mapping the top and bottom orientation of film of **3** with respect to the incident circularly polarized light (Figure 4F,G and Figure S43).

The large LDLB effect in the case of films of **2** and **3** could be due to the methoxy units of naproxen. Alkoxy groups attached to various aromatic cores have been shown to contribute to sample orientations with large LDLB responses [16,18,23]. This is in contrast with the negligible LDLB effect observed for SiPc-NDI **1**. The sidechain in **1** is more flexible and the aromatic unit attached on each side of NDI core can arrange differently with respect to the surface. 

**Figure 5:**
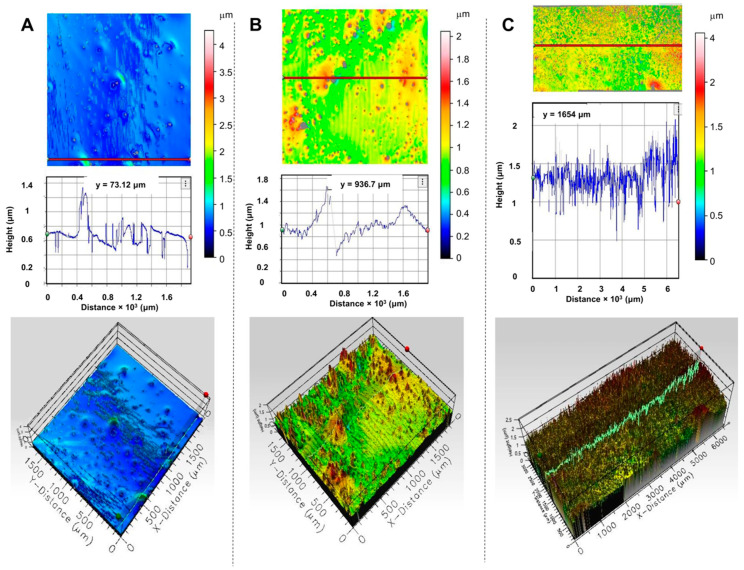
Images of SiPcs films deposited from TCE obtained with a profilometer showing the 2D top, cross section graphs, and 3D view maps: (**A**) film of **1**; (**B**) film of **2**; (**C**) film of **3**. The height scale is given for each film. The cross section (thickness variation) of each film was measured with the step height function of the profilometer instrument at the indicated point on the 2D maps (indicated by the horizontal red line).

The morphology and thickness uniformity of chiral SiPcs-based films have been assessed using the 3D profilometer available at DLS B23 (described in Method and Materials section). The 3D images of thin films of **1**–**3** from TCE are displayed in Figure 5. The morphologies of local domains of films show, in general, high coverage with good uniformity. The local thickness of the films varies between 1–2 μm, which is excellent given the drop-casting method used for deposition. Minor deviations (i.e., spikes) observed in the height of the films are also reflected in the CD*i* 2D maps. The film of SiPc-NDI (**1**) has the best local coverage across the 4 mm^2^ domain scanned and it is the thinnest (Figure 5A). The deposited material is homogeneously distributed across larger domains, as observed for an 18 mm^2^ scanned area of film of **3** (Figure 5C and Figure S45).

## 3. Materials and Methods

All reagents were purchased from commercial suppliers Merck (Gillingham, UK) and Thermo Fisher Scientific (Gloucester, UK) and Fluorochem (Derbyshire, UK), and they were used without further purification.

The microwave reactions were carried out in a CEM Discover microwave reactor (Buckingham, UK).

^1^H and ^13^C NMR spectra were recorded on 500 MHz Agilent Propulse (Stockport, UK) or 500 MHz Bruker Avance II+ (^1^H 500 MHz, ^13^C 126 MHz, Coventry, UK) instruments, as stated. Chemical shifts (δ) are reported in parts per million (ppm). Coupling constants are reported in Hertz (Hz), and signal multiplicity is denoted as multiplet (m), singlet (s), doublet (d), doublet of doublet (dd), triplet (t), quartet (q), sextet (sext). All spectra were acquired at 25 °C and were referenced to the residual solvent peaks. The ^1^H and ^13^C nuclear magnetic resonance (NMR) spectra have small traces of water and CH_2_Cl_2_.

Electrospray ionisation quadrupole time-of-flight (ESI-Q-TOF) mass spectrometry was performed on an Agilent Technologies 6545 Q-TOF LC-MS instrument (Stockport, UK) using a positive-ion mode.

All the CD/UV–vis data in solution was acquired on an Applied Photophysics Chirascan spectrophotometer (Leatherhead, UK) equipped with a Peltier temperature controller. Data was recorded in CH_2_Cl_2_ with a 1 cm pathlength fused silica cuvette; a background corresponding to cuvette/solvent absorption was subtracted from subsequent measurements. Settings: wavelength range 250–800 nm, time-per-point (dwell time) 1 sec, monochromator bandwidth 2 nm, temperature 20 °C. A Savitzky-Golay 9-point smoothing was applied to the ECD graph when processed.

ECD/UV–vis absorption solid state and CD*i* experiments were done at the B23 Synchrotron Radiation CD Beamline, Diamond Light Source, UK, using a nitrogen-flushed Modules A and B end-station spectrophotometer [16,23]. The film (in a 3D printed holder) was moved across different positions using Linkam MDS600 (Surrey, UK) with motorised XY stage [25]. The ECD/UV–vis absorption and 2D maps spectra were recorded with either Module A or Module B with a vertical sample chamber tower with the film coated on fused silica (Suprasil) substrate of 15 mm diameter and 1 mm thickness mounted on the motorised XY stage. The Olis GlobalWorks software (version 6.0) was used with the following parameters: wavelength range 200–450 nm, time-per-point (dwell time) 0.5 or 1 s, bandwidth of 2 nm, temperature 23 °C unless otherwise stated. The ECD spectrum of the fused silica substrate was measured as baseline and subtracted from those of the coated films. ECD/UV–vis absorption spectra were processed with Origin 2017 (version 9.4), while the 2D CD*i* maps were obtained with Excel. A Savitzky-Golay 9-point smoothing was applied to ECD/UV–vis absorption graphs when processed.

The 2D and 3D measurements of the coated films were obtained with Profilm3D (Filmetrics, San Diego, CA, USA) instrument available at B23, Diamond Light Source, UK and data processed using Profilm3D Optical Profilometer software (version 4.0.1.0).

Synthesis of **1:**

A 10-mL microwave tube was charged with silicon phthalocyanine dichloride (PcSi(Cl)_2_; 7.2 mg, 0.012 mmoles, 1 equiv), (*S*)-3-phenyl-2-(1,3,6,8-tetraoxo-7-((*S*)-2-phenylpropyl)-3,6,7,8- tetrahydrobenzo[lmn][3,8]phenanthrolin-2(1H)-yl)propanoic acid (*S*,*S*-NDI) (33 mg, 0.62 mmoles, 5 equiv), *N*,*N*-diisopropylethylamine (0.5 mL) and toluene (5 mL). The reaction mixture was heated under microwave irradiation for 14 h at 155 °C. The blue residue obtained after solvent evaporation under reduced pressure was redissolved in ethyl acetate (50 mL). The unreacted PcSi(Cl)*_2_* was filtered off, and the filtrate was washed with saturated NaHCO_3_ (3 × 50 mL) and water (3 × 50 mL). The organic fraction was dried over anhydrous MgSO_4_ and the solvent removed under reduced pressure. The crude residue was purified by column chromatography on silica-gel with CH_2_Cl_2_ to yield **1** as a green-blue solid (8.08 mg, 0.005 mmoles, 42%). ^1^H NMR (500 MHz, CDCl_3_): δ 9.21–9.16 (m, 8H), 8.39 (d, *J* = 7.4 Hz, 4H), 8.21–8.15 (m, 8H), 7.54 (d, *J* = 8.0 Hz, 4H), 7.49 (d, *J* = 7.4 Hz, 4H), 7.45 (t, *J* = 8.0 Hz, 4H), 7.33 (d, *J* = 7.3 Hz, 2H), 6.48 (t, *J* = 7.3 Hz, 2H), 6.42 (t, *J* = 7.3 Hz, 4H), 5.90 (d, *J* = 8.0 Hz, 4H), 4.67 (dd, *J* = 13.4, 8.1 Hz, 2H), 4.52 (dd, *J* = 13.4, 8.1 Hz, 2H), 3.71 (sext, *J* = 7.4 Hz, 2H), 2.86 (dd, *J* = 11.3, 4.7 Hz, 2H), 1.61 (dd, *J* = 15.4, 4.7 Hz, 2H), 1.58–1.53 (6H*), 1.52–1.48 (dd, *J* = 15.4, 4.7 Hz, 2H). * The chemical shift and multiplicity could not by determined due to the water peak. The ^1^H NMR spectrum is clean and matches the one reported in the literature [18].

Synthesis of **2:**

A 10-mL microwave tube was charged with silicon phthalocyanine dichloride (PcSi(Cl)_2_; 29.2 mg, 0.05 mmoles, 1 equiv), (*S*)-2-(6-methoxy-2-naphtyl) propionic acid (55 mg, 0.24 mmoles, 5 equiv), *N*,*N*-diisopropylethylamine (0.5 mL) and toluene (5 mL). The reaction mixture was heated under microwave irradiation for 14 h at 155 °C. The blue residue obtained after solvent evaporation under reduced pressure was redissolved in ethyl acetate (50 mL). The unreacted PcSi(Cl)_2_ was filtered off, and the filtrate was washed with saturated NaHCO_3_ (3 × 50 mL) and water (3 × 50 mL). The organic fraction was dried over anhydrous MgSO_4_ and the solvent removed under reduced pressure. The crude residue was purified by column chromatography on silica-gel with CH_2_Cl_2_:ethyl acetate 9:1 *v*/*v* to yield **2** as a green-blue solid (31.6 mg, 0.03 mmoles, 68%). ^1^H NMR (500 MHz, CDCl_3_) δ 9.40−9.38 (m, 8H), 8.32−8.28 (m, 8H), 7.06−7.00 (m, 2H), 6.88 (d, *J* = 8.7 Hz, 2H), 6.81 (d, *J* = 2.4 Hz, 2H), 6.50 (d, *J* = 8.2 Hz, 2H), 5.21 (s, 2H), 4.82 (dd, *J* = 8.3 Hz, 1.7 Hz, 2H), 4.09 (s, 6H), 0.55 (q, J = 6.9 Hz, 2H), −0.57 (d, J = 7.0 Hz, 6H). The ^1^H NMR spectrum is clean and matches the one reported in the literature [18].

Synthesis of **3:**

A 10-mL microwave tube was charged with silicon phthalocyanine dichloride (PcSi(Cl)_2_; 29.2 mg, 0.05 mmoles, 1 equiv), (*R*)-2-(6-methoxy-2-naphtyl) propionic acid (55 mg, 0.24 mmoles, 5 equiv), *N*,*N*-diisopropylethylamine (0.3 mL) and toluene (5 mL). The reaction mixture was heated under microwave irradiation for 14 h at 155 °C. The blue residue obtained after solvent evaporation under reduced pressure was redissolved in ethyl acetate (50 mL). The unreacted PcSi(Cl)_2_ was filtered off, and the filtrate was washed with saturated NaHCO_3_ (3 × 50 mL) and water (3 × 50 mL). The organic fraction was dried over anhydrous MgSO_4_ and the solvent removed under reduced pressure. The crude residue was purified by column chromatography on silica-gel with CH_2_Cl_2_:ethyl acetate 9:1 *v*/*v* to yield **3** as a green-blue solid (32.4 mg, 0.03 mmoles, 68%). ^1^H NMR (500 MHz, CDCl_3_) δ 9.42 (dd, *J* = 5.6, 3.0 Hz, 8H), 8.32 (dd, *J* = 5.6, 3.0 Hz, 8H), 7.06 (d, *J* = 8.8 Hz, 2H), 6.90 (d, *J* = 8.8 Hz, 2H), 6.84 (s, 2H), 6.53 (d, *J* = 8.2 Hz, 2H), 5.24 (s, 2H), 4.84 (d, *J* = 8.2 Hz, 2H), 4.11 (s, 6H), 0.58 (q, *J* = 6.9 Hz, 4H), −0.55 (d, *J* = 6.9 Hz, 6H). ^13^C NMR (126 MHz, CDCl_3_) δ 167.2, 156.9, 149.6, 135.2, 134.3, 132.5, 130.8, 128.9, 128.1, 125.8, 124.0, 123.7, 123.2, 118.2, 105.6, 55.4, 44.6, 15.5. ESI-Q-TOF *m/z* calculated for C_60_H_42_N_8_O_6_Si: 1021.2889 [M + Na]^+^; found 1021.2879. Copies of the NMR and UV-vis spectra can be found in Figures S1–S3.

Methodology for film deposition:

**Fused silica preparation:** fused silica plates of the following dimensions were used: length: 2.52–2.55 cm, width: 1.20–1.21 cm, height: 0.1 cm. The plates were cleaned by soaking them in concentrated nitric acid for 3–4 h, followed by individual rinsing with deionised water multiple times and subsequent soaking in concentrated nitric acid overnight (approximately 12 h). The plates were then thoroughly rinsed again one by one with deionised water multiple times, with a last wash using ultra-pure Milli-Q water and dried under a stream of nitrogen.

**Film deposition:** solutions for films were prepared by dissolving molecules **1**–**3** in TCE or DMF (molecule **1**) at a concentration of 10^−3^ M. A volume of 40 μL was deposited on the top side of each plate by the drop-casting method. The plates were then placed in a heated oven and kept for 30 min at 100 °C. All films were kept at room temperature at least 2 h before analysis.

## 4. Conclusions

Here, we reported the first SiPcs-based thin films with chiroptical properties obtained from chiral building blocks. We took advantage of highly collimated beamline and the CD*i* technique available at B23, the Diamond Light Source, to map the chiroptical properties of local domains of films at high spatial resolution unattainable with bench-top CD instruments. These results suggest that chiroptical properties of thin films of SiPcs can be controlled by the nature of the axial ligands and depend on the solvent used in the process of film preparation. Naproxen units attached to the SiPc core contribute to anisotropic assemblies with unprecedented, large LDLB effects important for the development of chiroptical sensors. The chiroptical properties of NDI-functionalized SiPc films are less influenced by sample orientation, consequently having low *g*-factors. These films are highly stable and robust, preserving their properties when exposed to temperature up to 80 °C and over long periods of time. The drop-cast method gives consistent results, allowing us to obtain homogeneous films with uniform thickness of 1–2 μm. This work puts forward a new approach of making chiral films based on versatile SiPcs for optoelectronic material applications.

## Supplementary Materials

The following are available online. Figure S1: ^1^H nuclear magnetic resonance (NMR) spectrum of **3** in CDCl_3_. Figure S2: ^13^C NMR spectrum of **3** in CDCl_3_. Figure S3. UV–vis absorption spectrum of **3** in CH_2_Cl_2_ (2 × 10^−5^ M). Figure S4. Overlaid local ECD spectra of thin films of **1** from TCE (red line) and DMF (blue line). Figure S5. Overlaid local ECD spectra of thin films of **1** freshly made from DMF (orange line) and the same film after being kept for 11 months at room temperature (blue line). Figure S6. Local ECD spectra of thin film of **2** from TCE, top (dashed line) and bottom (solid line). Figure S7. Local ECD spectra of thin film of **3** from TCE, top (dashed line) and bottom (solid line). Figure S8. Overlaid local UV–vis absorption spectra of films of **1** from TCE (red line) and from DMF (blue line). Figure S9. Overlaid local UV–vis absorption spectra of freshly made (orange line) and the 11-month-old (blue line) film of **1** from DMF. Figure S10. Local UV–vis absorption top (dashed line) and bottom (solid line) spectra of film of **2** from TCE. Figure S11. Local UV–vis absorption top (dashed line) and bottom (solid line) spectra of film of **3** from TCE. Figure S12. UV–vis absorption 2D map of freshly made film of **1** from DMF (25 × 25 grid array area of 0.2 mm step size at 391 nm; top side). Figure S13. ECD 2D map of freshly made film of **1** from DMF (25 × 25 grid array area of 0.2 mm step size at 391 nm; top side). Figure S14. Dissymmetry g-factor 2D map of freshly made film of **1** from DMF (25 × 25 grid array area of 0.2 mm step size at 391 nm; top side). Figure S15. UV–vis absorption 2D map of freshly made film of **1** from DMF (25 × 25 grid array area of 0.2 mm step size at 237 nm; top side). Figure S16. ECD 2D map of freshly made film of **1** from DMF (25 × 25 grid array area of 0.2 mm step size at 237 nm; top side). Figure S17. Dissymmetry g-factor 2D map of freshly made film of **1** from DMF (25 × 25 grid array area of 0.2 mm step size at 237 nm; top side). Figure S18. UV–vis absorption 2D map of a 11 months old film of **1** from DMF (25 × 25 grid array area of 0.2 mm step size at 391 nm; top side). Figure S19. ECD 2D map of an 11-month-old film of **1** from DMF (25 × 25 grid array area of 0.2 mm step size at 391 nm; top side). Figure S20. Dissymmetry g-factor 2D map of an 11-month-old film of **1** from DMF (25 × 25 grid array area of 0.2 mm step size at 391 nm; top side). Figure S21. UV–vis absorption 2D map of an 11-month-old film of **1** from DMF (25 × 25 grid array area of 0.2 mm step size at 391 nm; bottom side). Figure S22. ECD 2D map of an 11-month-old film of **1** from DMF (25 × 25 grid array area of 0.2 mm step size at 391 nm; bottom side). Figure S23. Dissymmetry g-factor 2D map of a 11 months old film of **1** from DMF (25 × 25 grid array area of 0.2 mm step size at 391 nm; bottom side). Figure S24. UV–vis absorption 2D map of an 11-month-old film of **1** from DMF (25 × 25 grid array area of 0.2 mm step size at 237 nm; top side). Figure S25. ECD 2D map of an 11-month-old film of **1** from DMF (25 × 25 grid array area of 0.2 mm step size at 237 nm; top side). Figure S26. Dissymmetry g-factor 2D map of an 11-month-old film of **1** from DMF (25 × 25 grid array area of 0.2 mm step size at 237 nm; top side). Figure S27. UV–vis absorption 2D map of an 11-month-old film of **1** from DMF (25 × 25 grid array area of 0.2 mm step size at 237 nm; bottom side). Figure S28. ECD 2D map of an 11-month-old film of **1** from DMF (25 × 25 grid array area of 0.2 mm step size at 237 nm; bottom side). Figure S29. Dissymmetry g-factor 2D map of an 11-month-old film of **1** from DMF (25 × 25 grid array area of 0.2 mm step size at 237 nm; bottom side). Figure S30. VT ECD 2D CDi maps of top side of film of **1** at the specified temperatures (4 × 4 grid array area of 1 mm step size at 387 nm). Figure S31. UV–vis absorption 2D map of film of **1** from TCE (25 × 25 grid array area of 0.2 mm step size at 391 nm; top side). Figure S32. ECD 2D map of film of **1** from TCE (25 × 25 grid array area of 0.2 mm step size at 391 nm; top side). Figure S33. Dissymmetry g-factor 2D map of film of **1** from TCE (25 × 25 grid array area of 0.2 mm step size at 391 nm; top side). Figure S34. UV–vis absorption 2D map of film of **1** from TCE (25 × 25 grid array area of 0.2 mm step size at 237 nm; top side). Figure S35. ECD 2D map of film of **1** from TCE (25 × 25 grid array area of 0.2 mm step size at 237 nm; top side). Figure S36. Dissymmetry g-factor 2D map of film of **1** from TCE (25 × 25 grid array area of 0.2 mm step size at 237 nm; top side). Figure S37. UV–vis absorption 2D map of **2** of film from TCE (25 × 25 grid array area of 0.2 mm step size at 391 nm; top side). Figure S38. ECD 2D map of **2** of film from TCE (25 × 25 grid array area of 0.2 mm step size at 391 nm; top side). Figure S39. Dissymmetry g-factor 2D map of **2** of film from TCE (25 × 25 grid array area of 0.2 mm step size at 391 nm; top side). Figure S40. UV–vis absorption 2D map of **2** of film from TCE (25 × 25 grid array area of 0.2 mm step size at 311 nm; top side). Figure S41. ECD 2D map of **2** of film from TCE (25 × 25 grid array area of 0.2 mm step size at 311 nm; top side). Figure S42. Dissymmetry g-factor 2D map of **2** of film from TCE (25 × 25 grid array area of 0.2 mm step size at 311 nm; top side). Figure S43. ECD 2D CDi maps of film of **3** from TCE (7 × 7 grid array area of 0.2 mm step size) (**a**) top side at 391 nm, (**b**) bottom side at 391 nm, (**c**) top side at 311 nm, (**d**) bottom side at 311 nm. Figure S44. 3D g-factor maps of film of **1** from DMF at 391 nm: (**a**) top, (**b**) bottom, (**c**) bottom flipped along the *y*-axis, indicating good degree of homogeneity and lack of LDLB contributions. Figure S45. Thickness-related measured by absorption of film of **1** from DMF; x- and y-axes are represented at 0.2 mm steps, while the *z*-axis is represented in Absorbance units.
