# Supplementary material for: Mapping the Chiroptical Properties of Local Domains in Thin Films of Chiral Silicon Phthalocyanines by CD Imaging
Source: Molecules. 2020 Dec 21;25(24):6048. doi: 10.3390/molecules25246048 (PMC7767354; doi:10.3390/molecules25246048)
Supplement: Supplementary file 1 [file molecules-25-06048-s001.pdf]

Supplementary Materials for

Mapping the chiroptical properties of local domains in thin films of chiral silicon phthalocyanines by CD imaging

Dora-Maria Răsădean, Tiberiu-Marius Gianga, Tamás Jávorfi, Rohanah Hussain, Giuliano Siligardi and G. Dan Pantoș

Correspondence to: g.d.pantos@bath.ac.uk

Supplementary Figures S1-S45

Table of Contents

1. Spectroscopic characterisation of molecule 3 3

Fig. S1: ^1H NMR spectrum of 3 in $CDCl_3$. 3

Fig. S2: ^{13}C NMR spectrum of 3 in $CDCl_3$. 3

Fig. S3. UV-vis absorption spectrum of 3 in CH_2Cl_2 4

2. ECD/UV-vis absorption spectra of thin films of 1 – 3 4

Fig. S4. Overlaid local ECD spectra of thin films of 1 from TCE and DMF 4

Fig. S5. Overlaid local ECD spectra of thin films of 1 freshly made from DMF and the same film after kept 11 months at room temperature 5

Fig. S6. Local ECD spectra of thin film of 2 from TCE. 5

Fig. S7. Local ECD spectra of thin film of 3 from TCE. 6

Fig. S8. Overlaid local UV-vis absorption spectra of films of 1 from TCE and from DMF. 6

Fig. S9. Overlaid local UV-vis absorption spectra of freshly made and the 11 months old film of 1 from DMF 7

Fig. S10. Local UV-vis absorption spectra of film of 2 from TCE 7

Fig. S11. Local UV-vis absorption spectra of film of 3 from TCE 8

3. 2D CDi maps of films of 1 – 3 8

Fig. S12. UV-vis absorption 2D map of freshly made film of 1 from DMF 8

Fig. S13. ECD 2D map of freshly made film of 1 from DMF 9

Fig. S14. Dissymmetry g-factor 2D map of freshly made film of 1 from DMF 9

Fig. S15. UV-vis absorption 2D map of freshly made film of 1 from DMF 10
Fig. S16. ECD 2D map of freshly made film of 1 from DMF 10
Fig. S17. Dissymmetry g-factor 2D map of freshly made film of 1 from DMF 11
Fig. S18. UV-vis absorption 2D map of a 11 months old film of 1 from DMF 11
Fig. S19. ECD 2D map of a 11 months old film of 1 from DMF 12
Fig S20. Dissymmetry g-factor 2D map of a 11 months old film of 1 from DMF 12
Fig. S21. UV-vis absorption 2D map of a 11 months old film of 1 from DMF 13
Fig. S22. ECD 2D map of a 11 months old film of 1 from DMF 13
Fig. S23. Dissymmetry g-factor 2D map of a 11 months old film of 1 from DMF 14
Fig. S24. UV-vis absorption 2D map of a 11 months old film of 1 from DMF 14
Fig. S25. ECD 2D map of a 11 months old film of 1 from DMF 15
Fig. S26. Dissymmetry g-factor 2D map of a 11 months old film of 1 from DMF 15
Fig. S27. UV-vis absorption 2D map of a 11 months old film of 1 from DMF 16
Fig. S28. ECD 2D map of a 11 months old film of 1 from DMF 16
Fig. S29. Dissymmetry g-factor 2D map of a 11 months old film of 1 from DMF 17
Fig. S30. VT ECD 2D CDi maps of top side of film of 1 at the specified temperatures 17
Fig. S31. UV-vis absorption 2D map of film of 1 from TCE 18
Fig. S32. ECD 2D map of film of 1 from TCE 18
Fig. S33. Dissymmetry g-factor 2D map of film of 1 from TCE 19
Fig. S34. UV-vis absorption 2D map of film of 1 from TCE 19
Fig. S35. ECD 2D map of film of 1 from TCE 20
Fig. S36. Dissymmetry g-factor 2D map of film of 1 from TCE 20
Fig. S37. UV-vis absorption 2D map of 2 of film from TCE 21
Fig. S38. ECD 2D map of 2 of film from TCE 21
Fig. S39. Dissymmetry g-factor 2D map of 2 of film from TCE 22
Fig. S40. UV-vis absorption 2D map of 2 of film from TCE 22
Fig. S41. ECD 2D map of 2 of film from TCE 23
Fig. S42. Dissymmetry g-factor 2D map of 2 of film from TCE 23
Fig. S43. ECD 2D CDi maps of film of 3 from TCE 24
4. 3D map of film of 1 .... *25*
Fig. S44. 3D g-factor maps of film of 1 from DMF at 391 nm 25
5. Thickness of film of 1 .... *26*
Fig. S45. Thickness-related measured by absorption of film of 1 from DMF 26

1. Spectroscopic characterisation of molecule 3

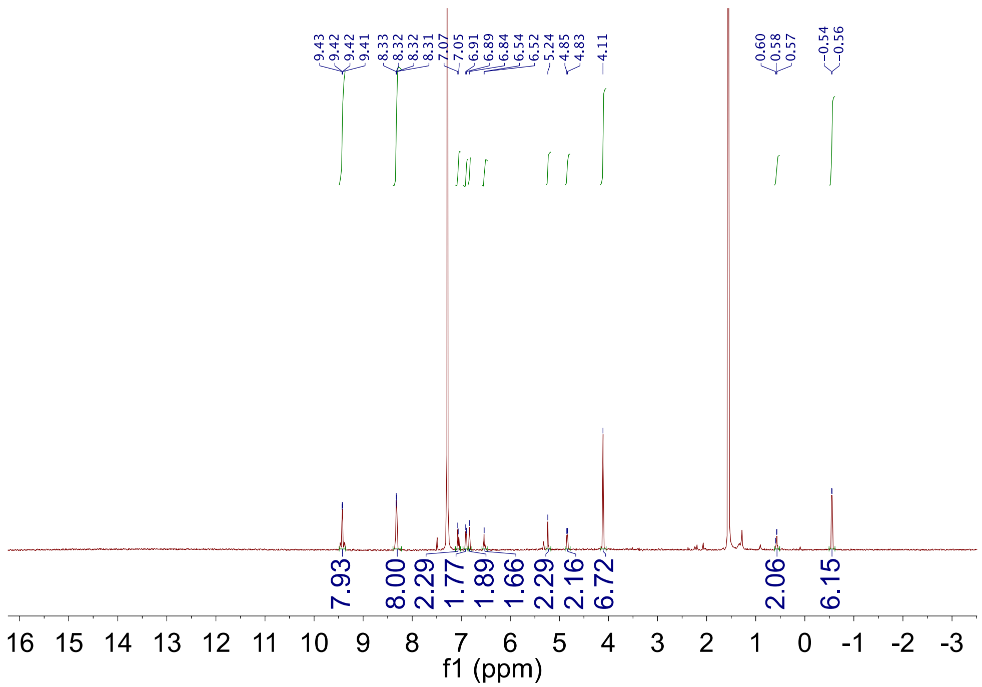

Fig. S1: ^{1}H NMR spectrum of 3 in $CDCl_3$.

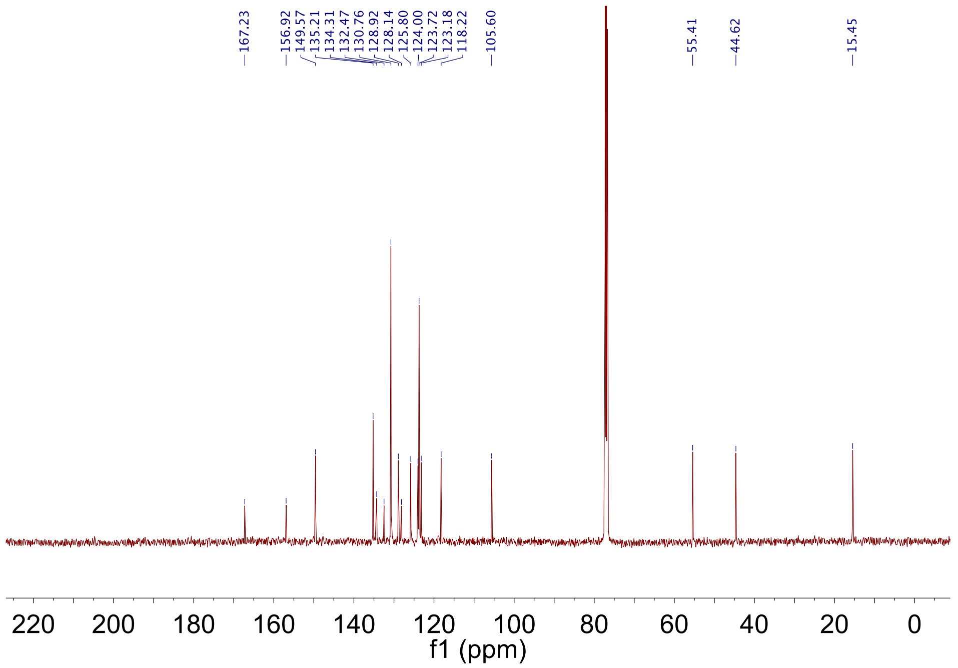

Fig. S2: ^{13}C NMR spectrum of 3 in $CDCl_3$.

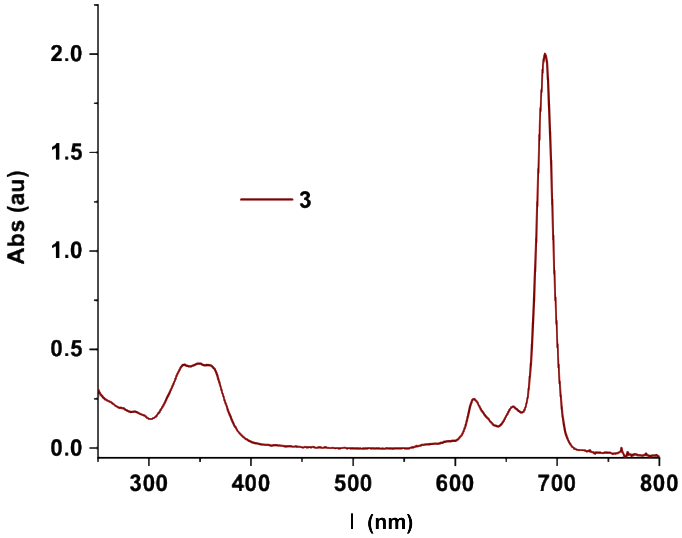

Fig. S3. UV-vis absorption spectrum of 3 in CH_2Cl_2 (2×10^{-5} M).

2. ECD/UV-vis absorption spectra of thin films of 1 – 3

All spectra of films not provided in the main manuscript are given below:

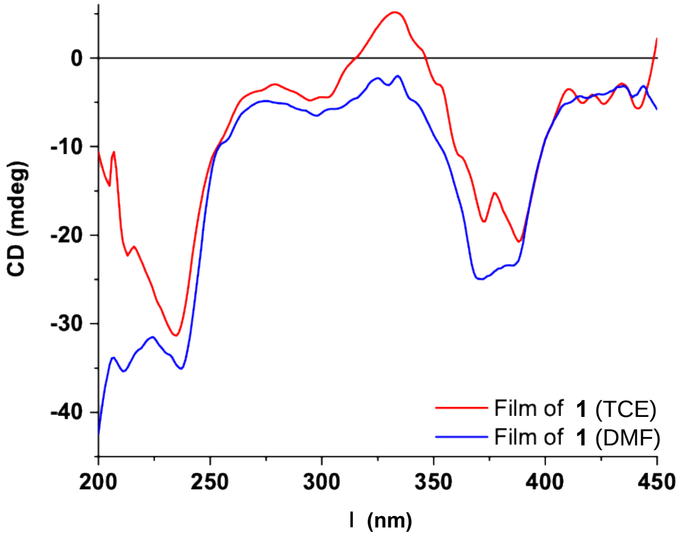

Fig. S4. Overlaid local ECD spectra of thin films of 1 from TCE (red line) and DMF (blue line).

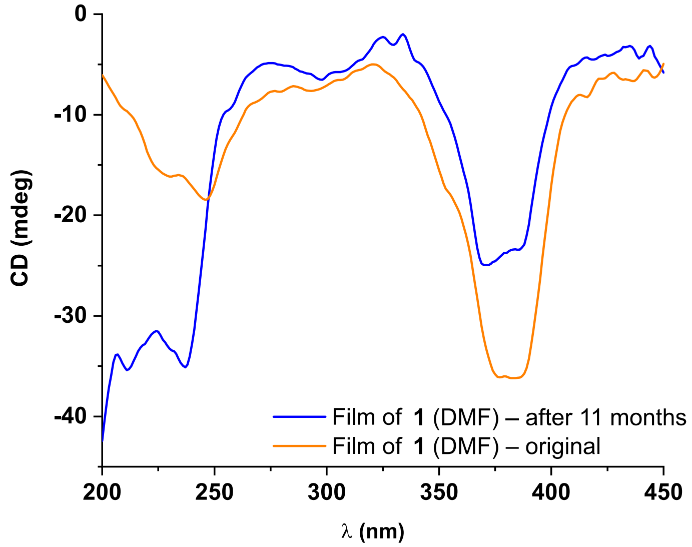

Fig. S5. Overlaid local ECD spectra of thin films of 1 freshly made from DMF (orange line) and the same film after kept 11 months at room temperature (blue line).

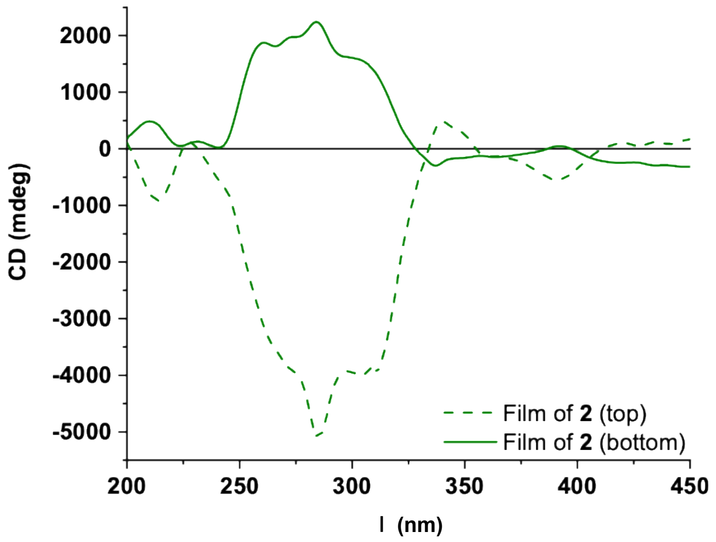

Fig. S6. Local ECD spectra of thin film of 2 from TCE, top (dashed line) and bottom (solid line).

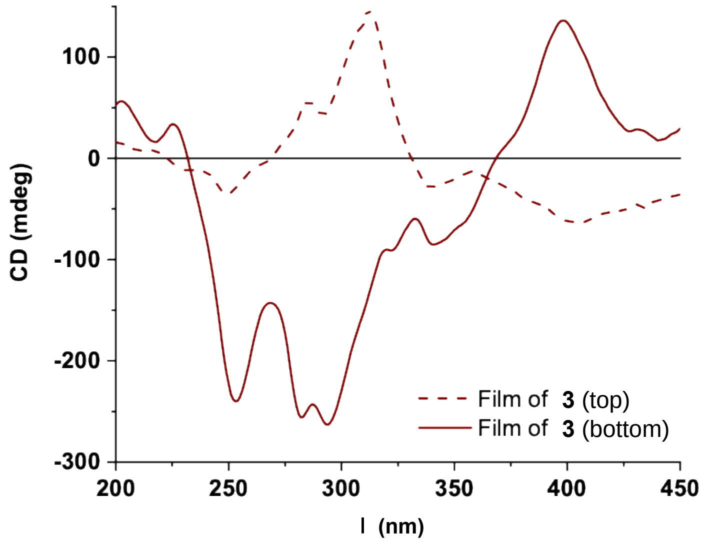

Fig. S7. Local ECD spectra of thin film of 3 from TCE, top (dashed line) and bottom (solid line).

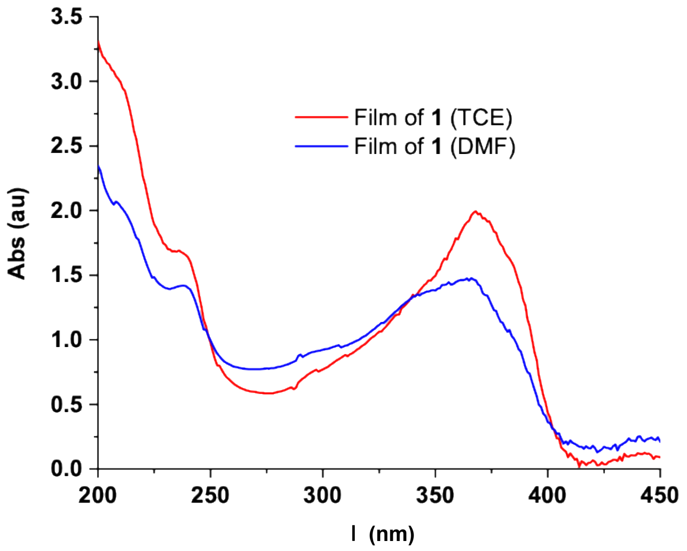

Fig. S8. Overlaid local UV-vis absorption spectra of films of 1 from TCE (red line) and from DMF (blue line).

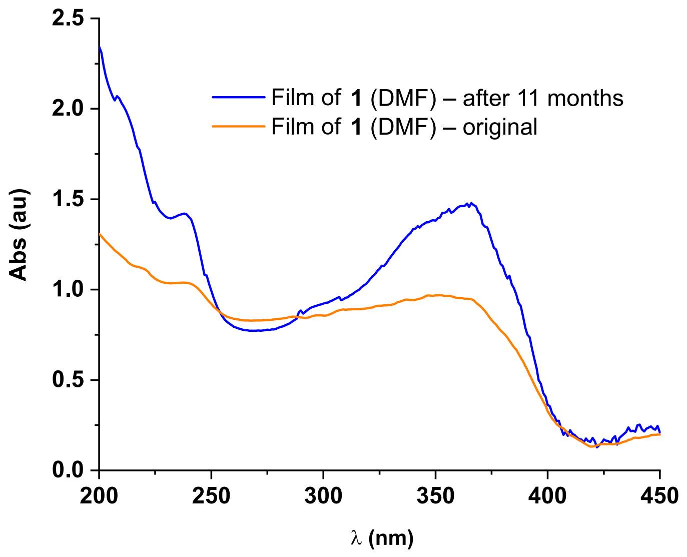

Fig. S9. Overlaid local UV-vis absorption spectra of freshly made (orange line) and the 11 months old (blue line) film of 1 from DMF.

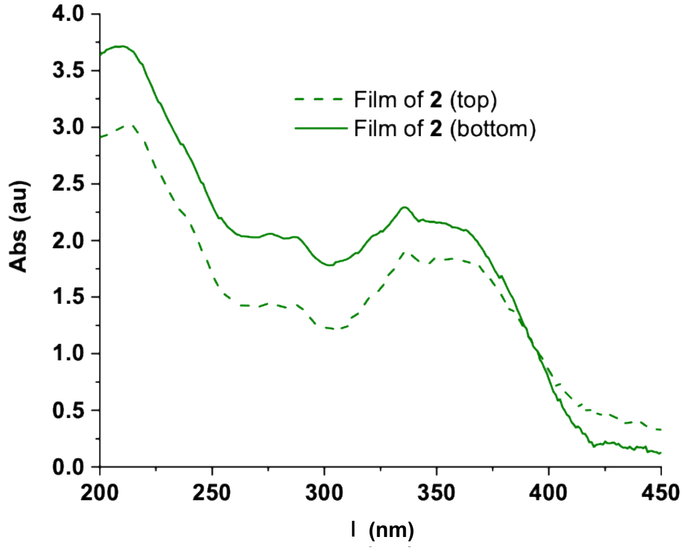

Fig. S10. Local UV-vis absorption top (dashed line) and bottom (solid line) spectra of film of 2 from TCE.

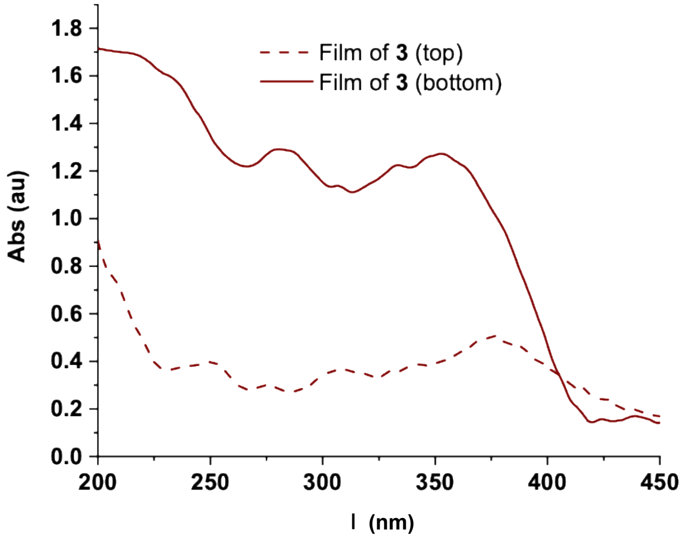

Fig. S11. Local UV-vis absorption top (dashed line) and bottom (solid line) spectra of film of 3 from TCE.

3. 2D CD*i* maps of films of 1 – 3

The mapped grid array area of each map is given in the corresponding caption.

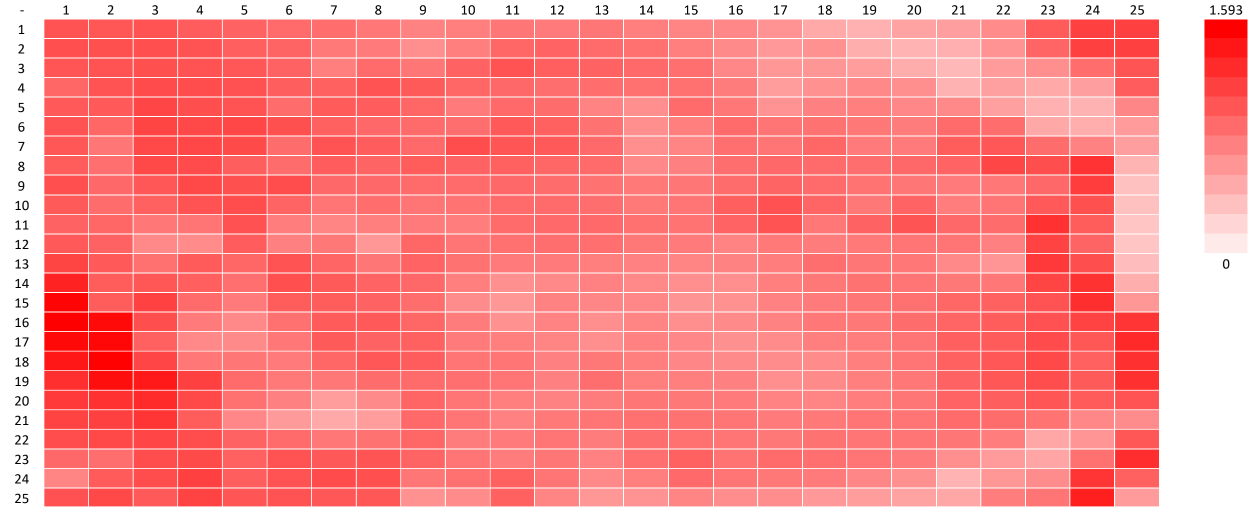

Fig. S12. UV-vis absorption 2D map of freshly made film of 1 from DMF (25 × 25 grid array area of 0.2 mm step size at 391 nm; top side).

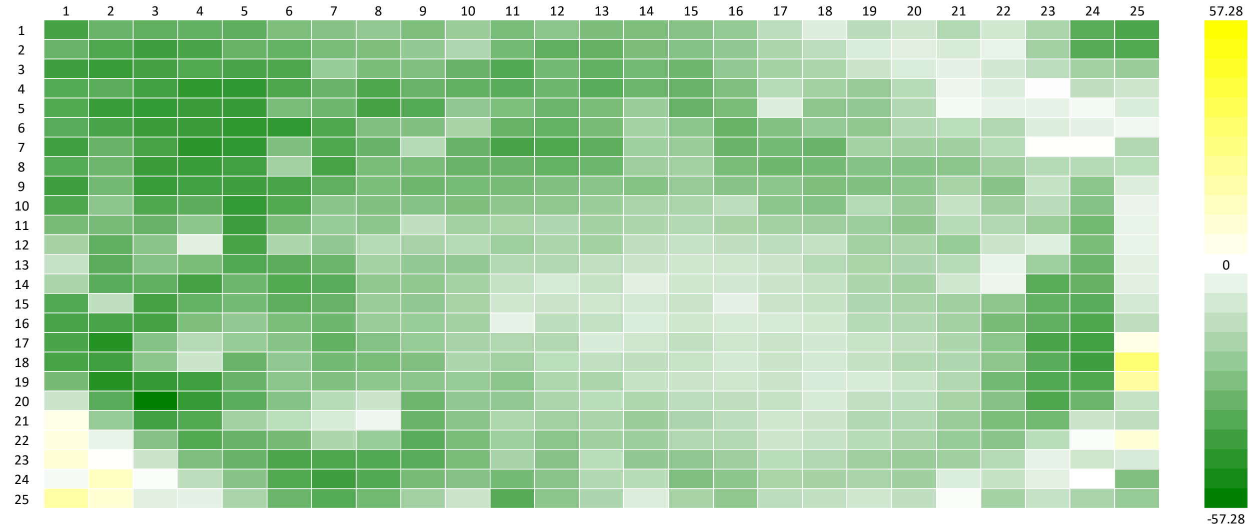

Fig. S13. ECD 2D map of freshly made film of 1 from DMF (25 × 25 grid array area of 0.2 mm step size at 391 nm; top side).

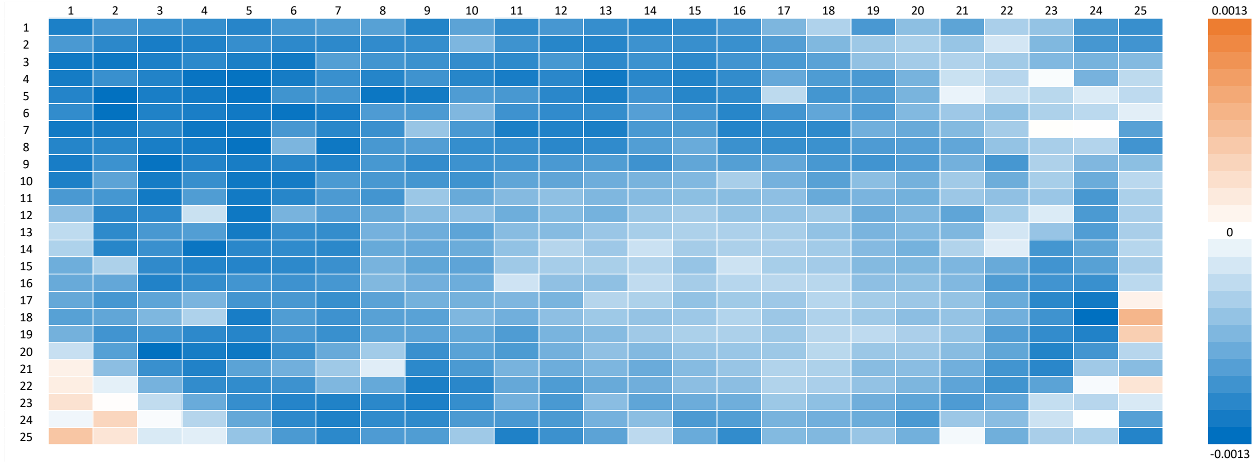

Fig. S14. Dissymmetry g-factor 2D map of freshly made film of 1 from DMF (25 × 25 grid array area of 0.2 mm step size at 391 nm; top side).

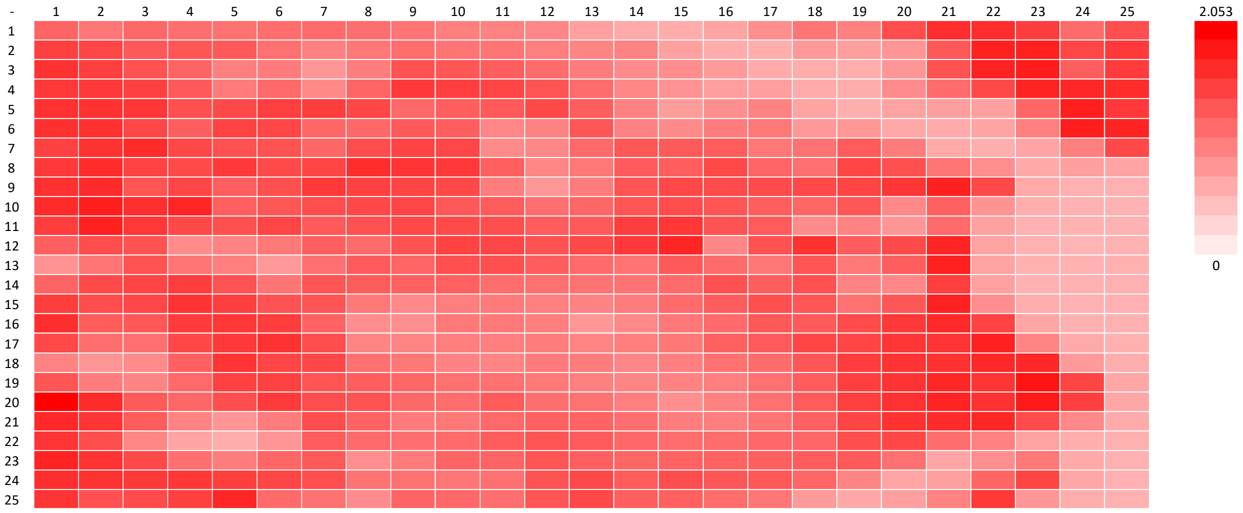

Fig. S15. UV-vis absorption 2D map of freshly made film of 1 from DMF (25 × 25 grid array area of 0.2 mm step size at 237 nm; top side).

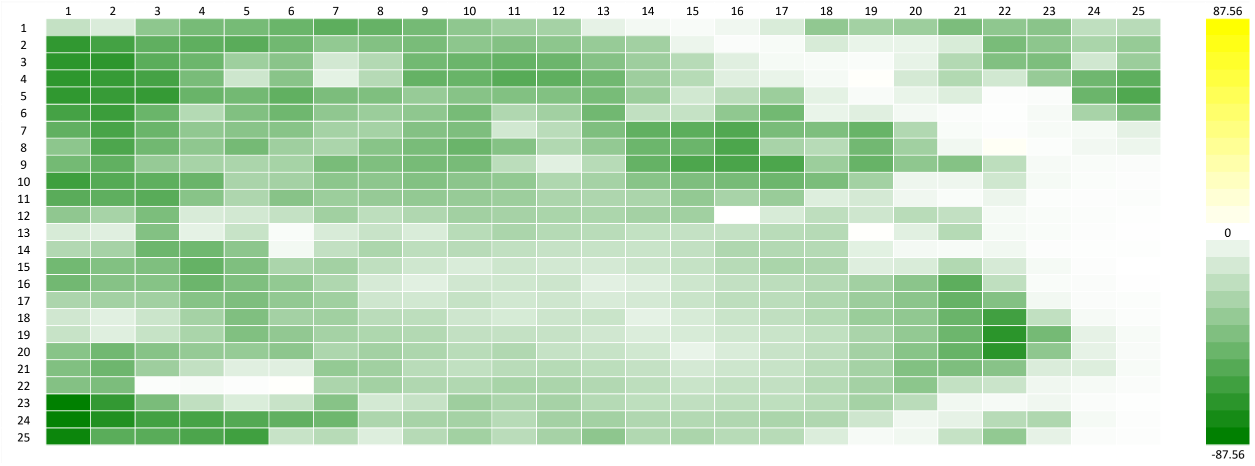

Fig. S16. ECD 2D map of freshly made film of 1 from DMF (25 × 25 grid array area of 0.2 mm step size at 237 nm; top side).

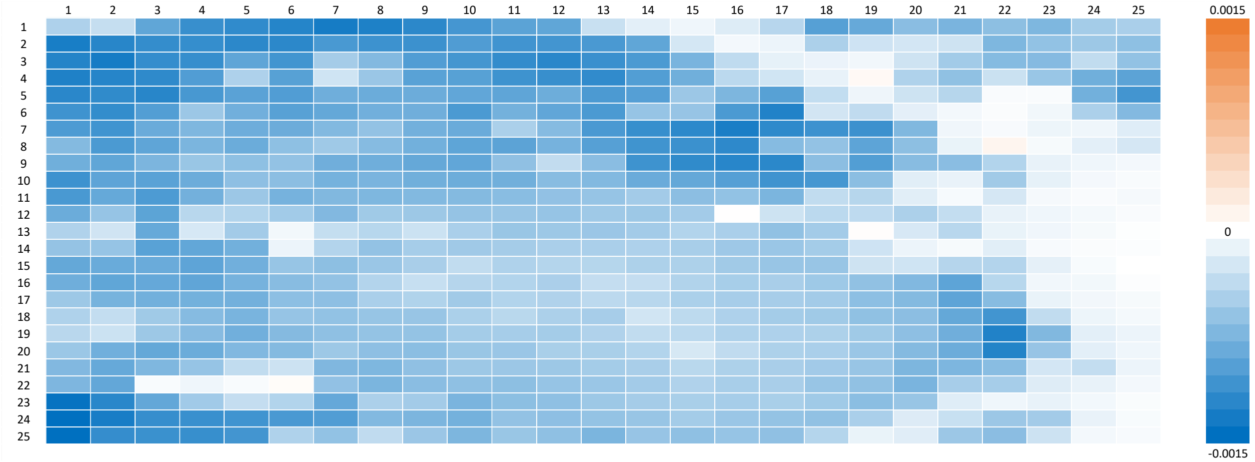

Fig. S17. Dissymmetry g-factor 2D map of freshly made film of 1 from DMF (25 × 25 grid array area of 0.2 mm step size at 237 nm; top side).

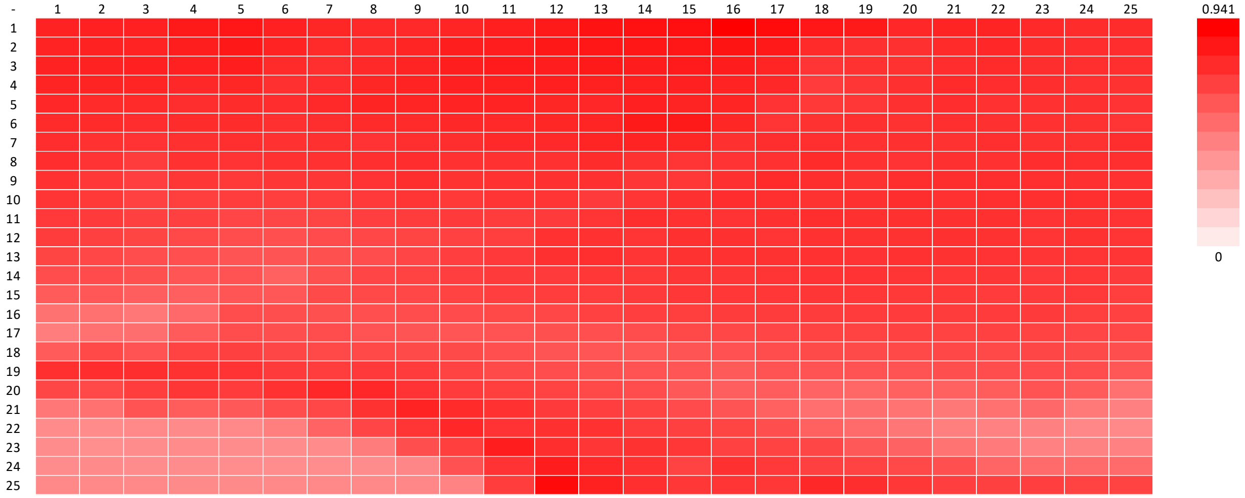

Fig. S18. UV-vis absorption 2D map of a 11 months old film of 1 from DMF (25 × 25 grid array area of 0.2 mm step size at 391 nm; top side).

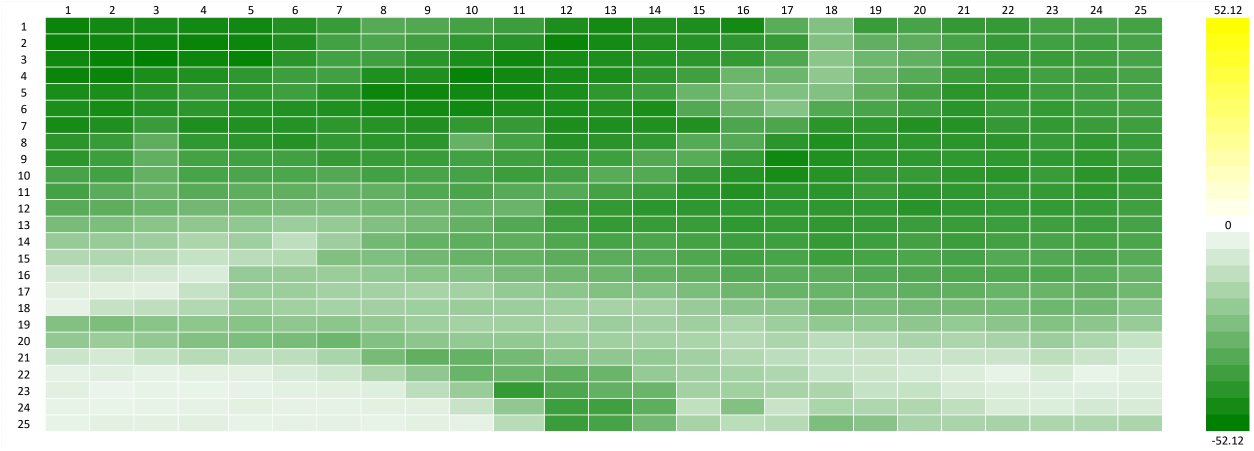

Fig. S19. ECD 2D map of a 11 months old film of 1 from DMF (25 × 25 grid array area of 0.2 mm step size at 391 nm; top side).

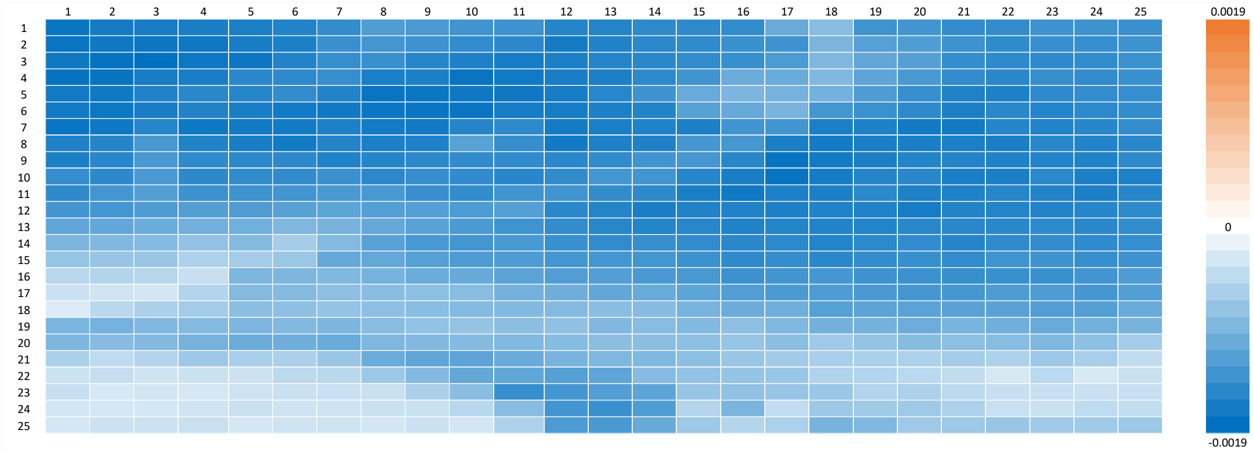

Fig S20. Dissymmetry g-factor 2D map of a 11 months old film of 1 from DMF (25 × 25 grid array area of 0.2 mm step size at 391 nm; top side).

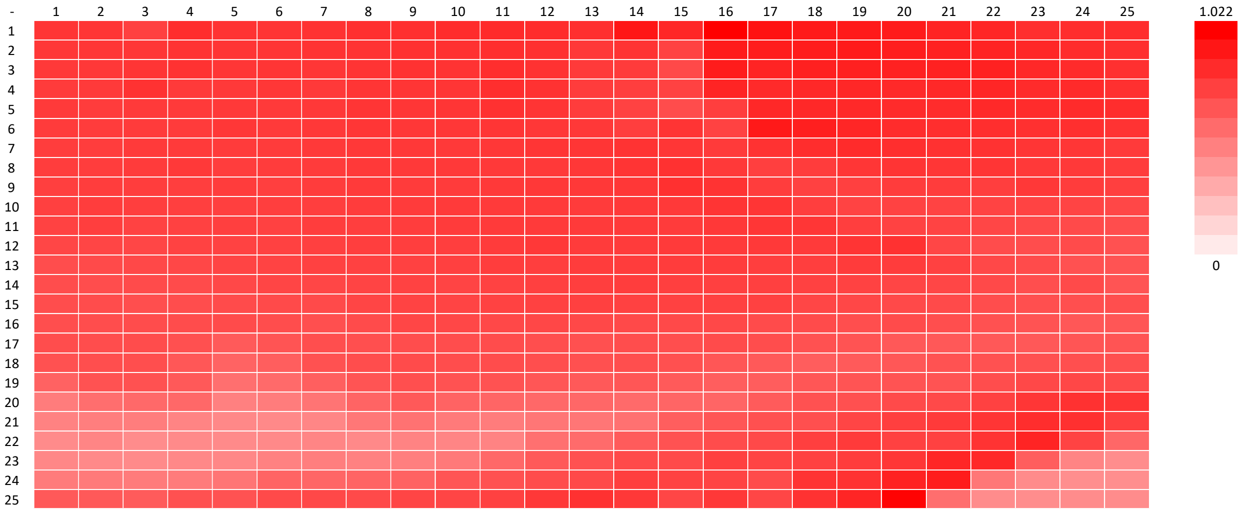

Fig. S21. UV-vis absorption 2D map of a 11 months old film of 1 from DMF (25 × 25 grid array area of 0.2 mm step size at 391 nm; bottom side).

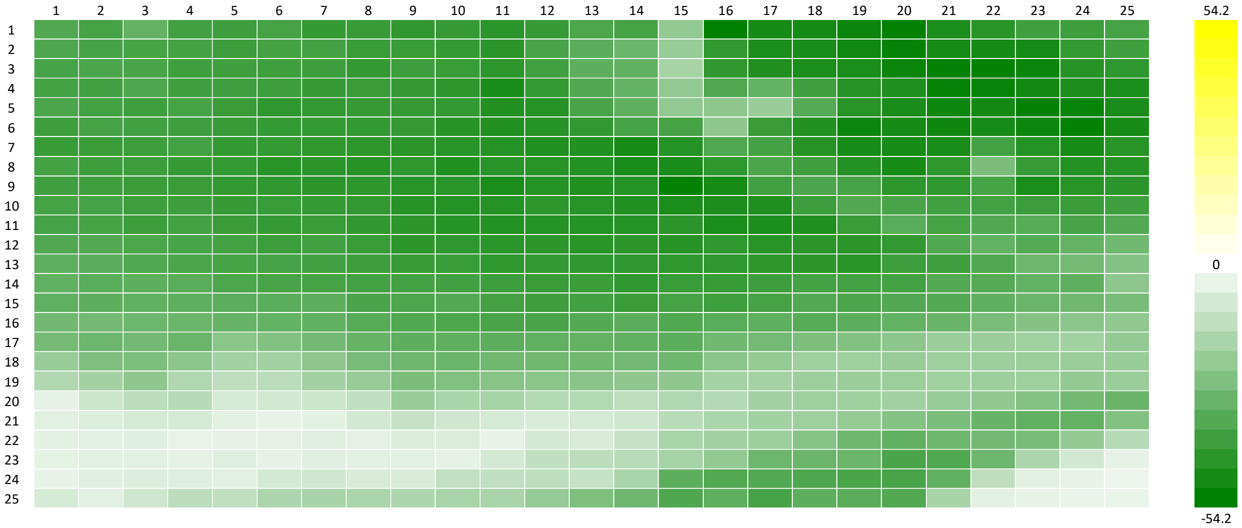

Fig. S22. ECD 2D map of a 11 months old film of 1 from DMF (25 × 25 grid array area of 0.2 mm step size at 391 nm; bottom side).

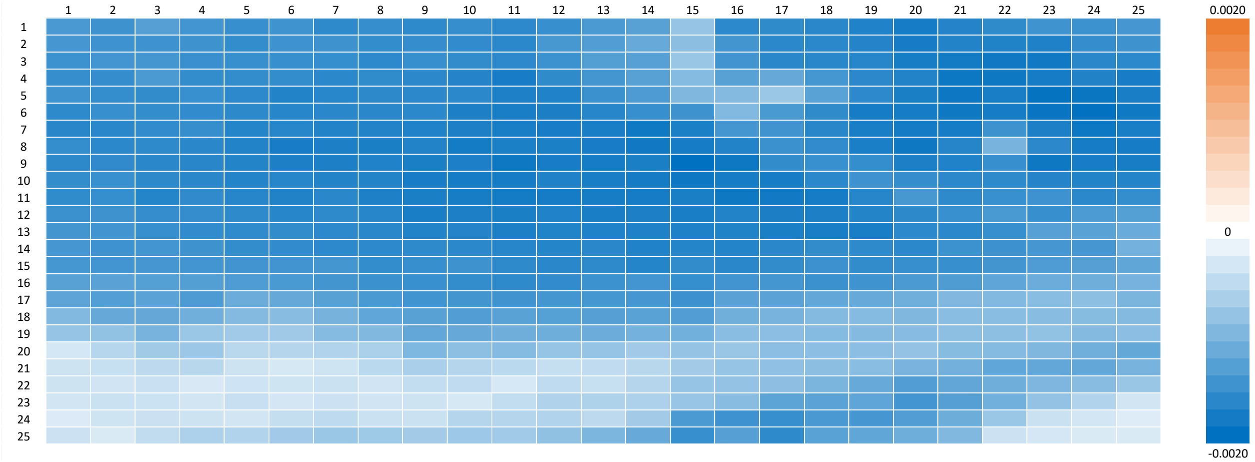

Fig. S23. Dissymmetry g-factor 2D map of a 11 months old film of 1 from DMF (25 × 25 grid array area of 0.2 mm step size at 391 nm; bottom side).

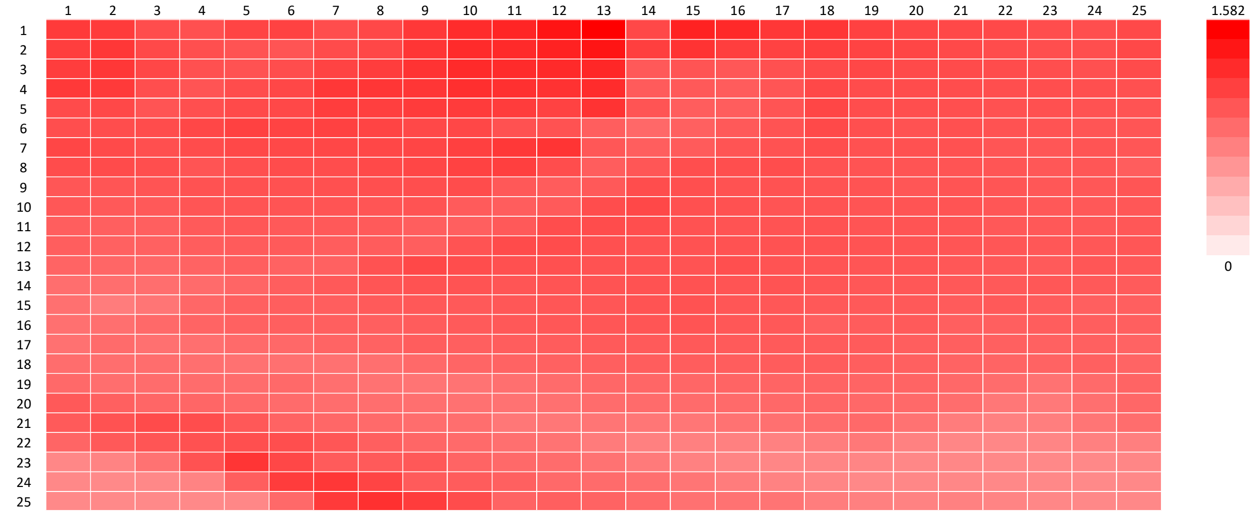

Fig. S24. UV-vis absorption 2D map of a 11 months old film of 1 from DMF (25 × 25 grid array area of 0.2 mm step size at 237 nm; top side).

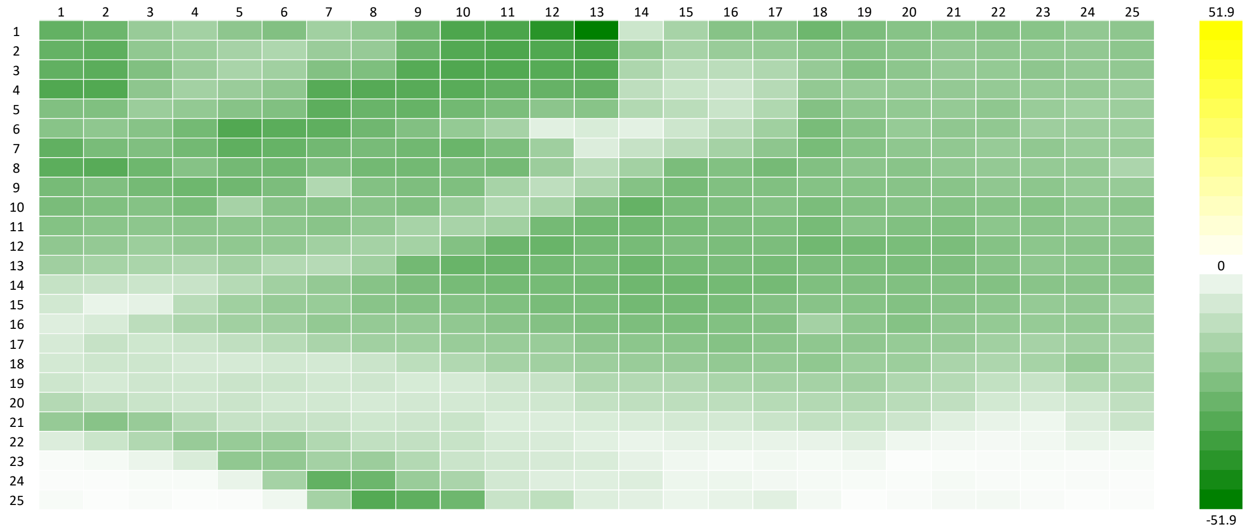

Fig. S25. ECD 2D map of a 11 months old film of 1 from DMF (25 × 25 grid array area of 0.2 mm step size at 237 nm; top side).

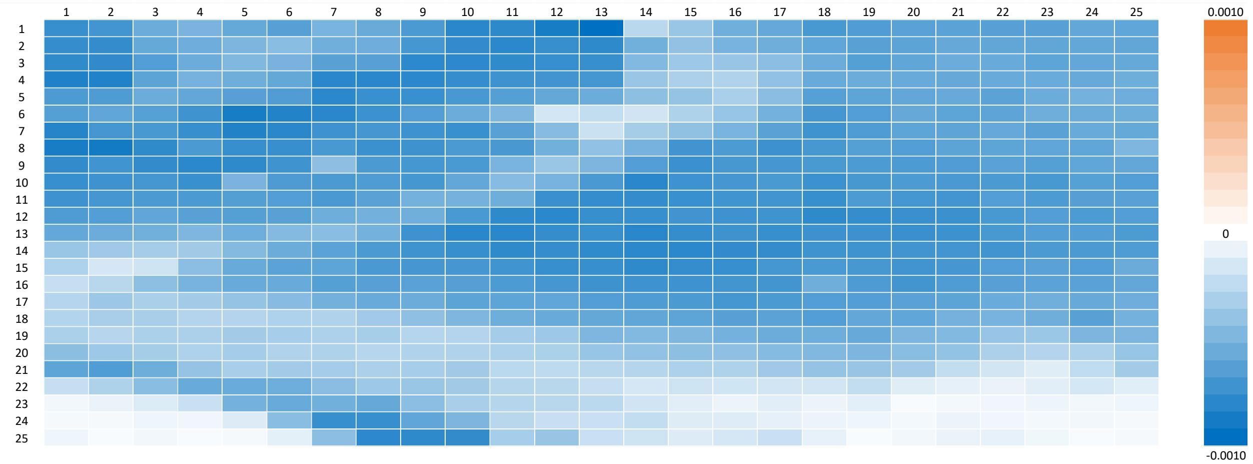

Fig. S26. Dissymmetry g-factor 2D map of a 11 months old film of 1 from DMF (25 × 25 grid array area of 0.2 mm step size at 237 nm; top side).

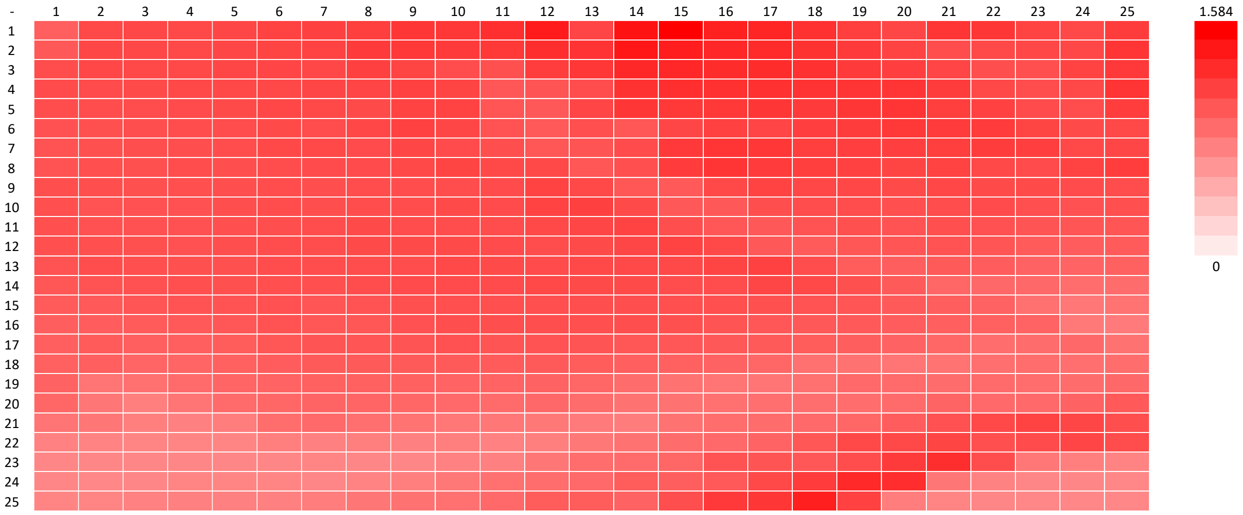

Fig. S27. UV-vis absorption 2D map of a 11 months old film of 1 from DMF (25 × 25 grid array area of 0.2 mm step size at 237 nm; bottom side).

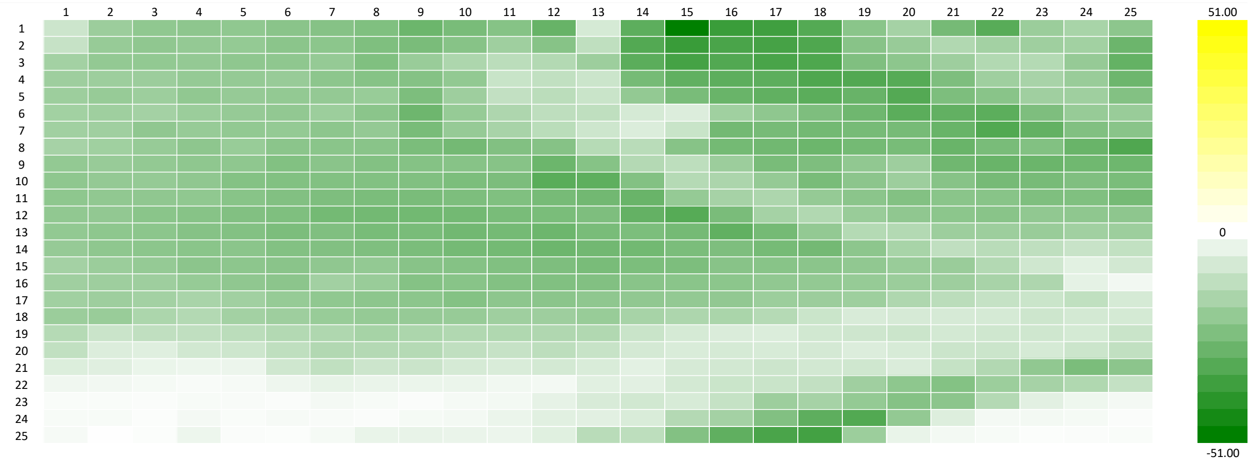

Fig. S28. ECD 2D map of a 11 months old film of 1 from DMF (25 × 25 grid array area of 0.2 mm step size at 237 nm; bottom side).

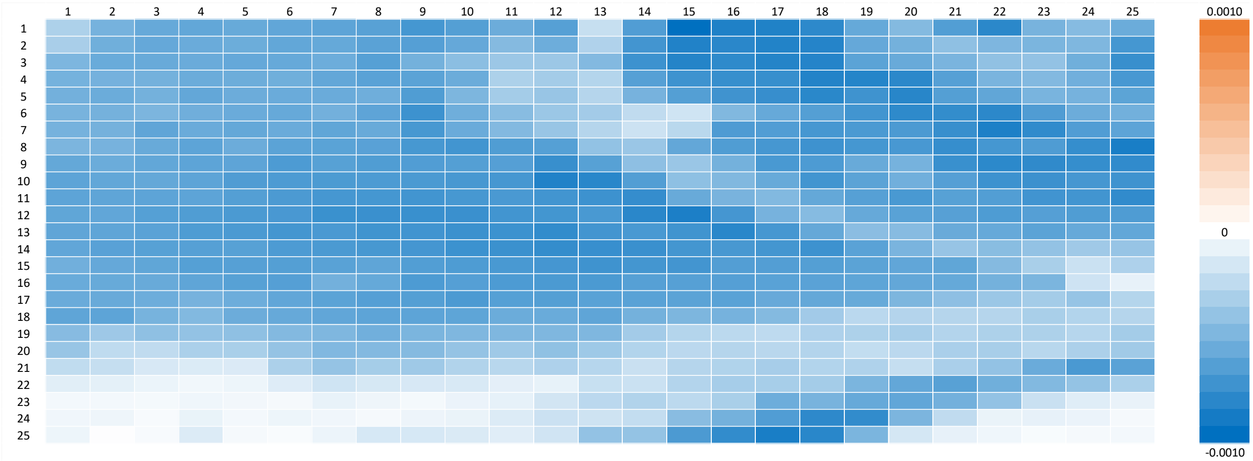

Fig. S29. Dissymmetry g-factor 2D map of a 11 months old film of 1 from DMF (25 × 25 grid array area of 0.2 mm step size at 237 nm; bottom side).

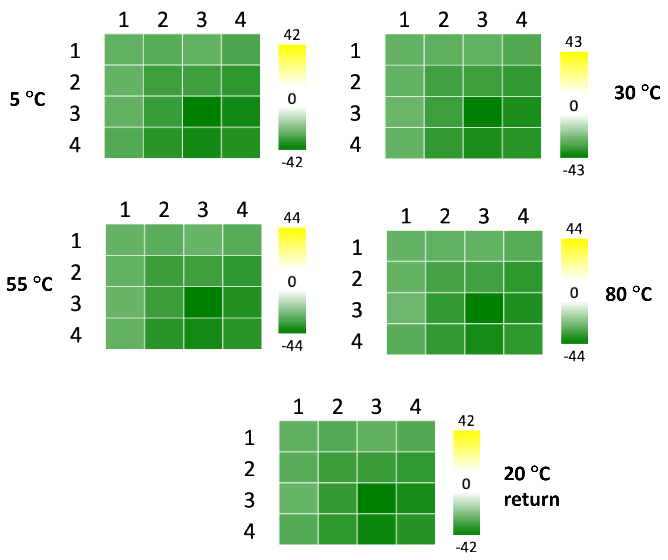

Fig. S30. VT ECD 2D CDi maps of top side of film of 1 at the specified temperatures (4 × 4 grid array area of 1 mm step size at 387 nm).

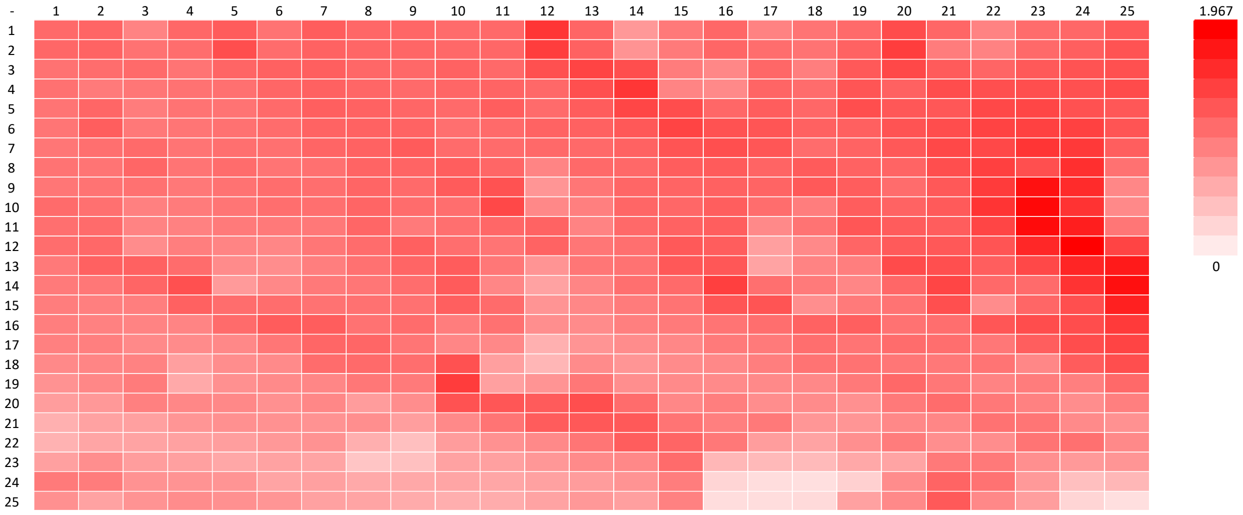

Fig. S31. UV-vis absorption 2D map of film of 1 from TCE (25 × 25 grid array area of 0.2 mm step size at 391 nm; top side).

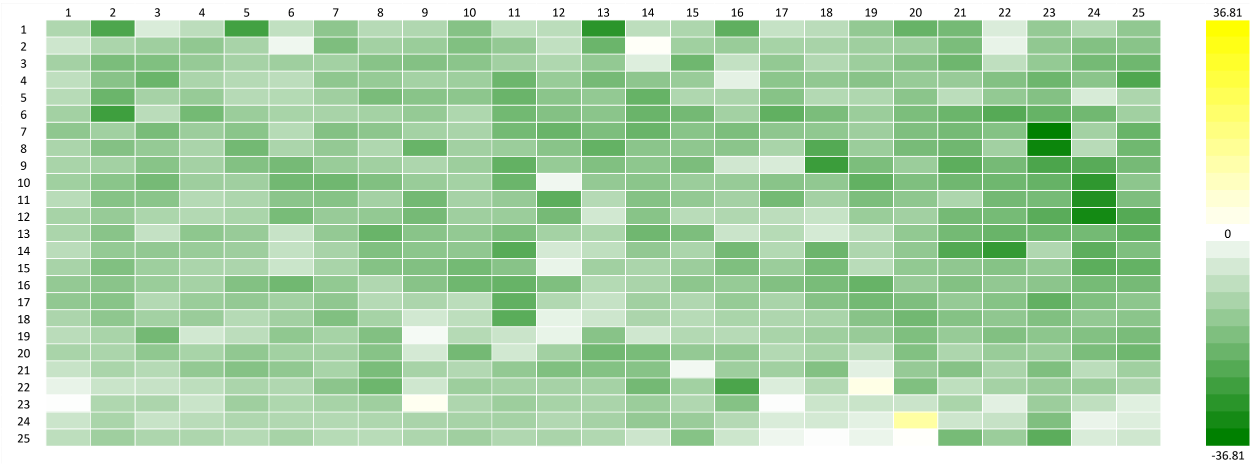

Fig. S32. ECD 2D map of film of 1 from TCE (25 × 25 grid array area of 0.2 mm step size at 391 nm; top side).

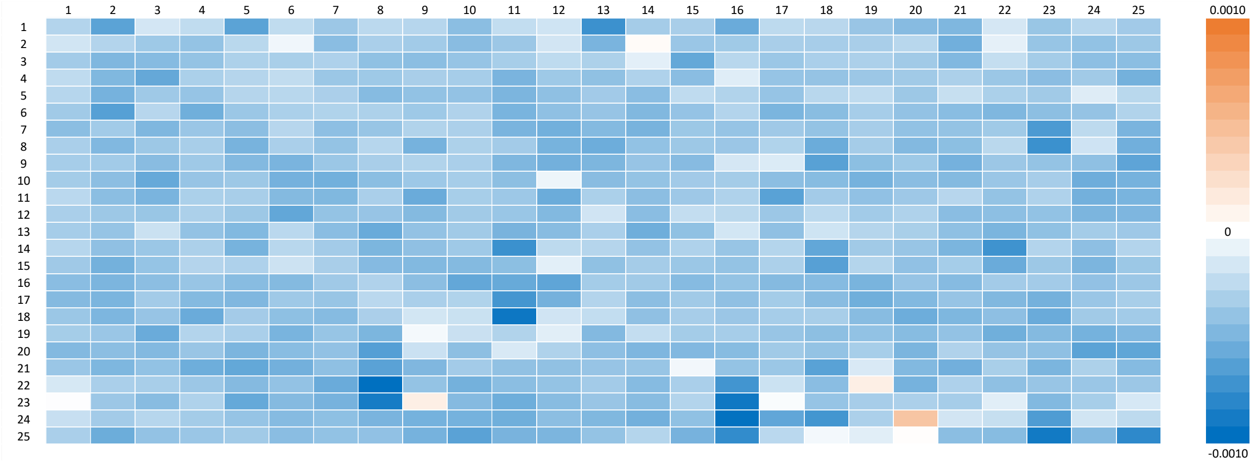

Fig. S33. Dissymmetry g-factor 2D map of film of 1 from TCE (25 × 25 grid array area of 0.2 mm step size at 391 nm; top side).

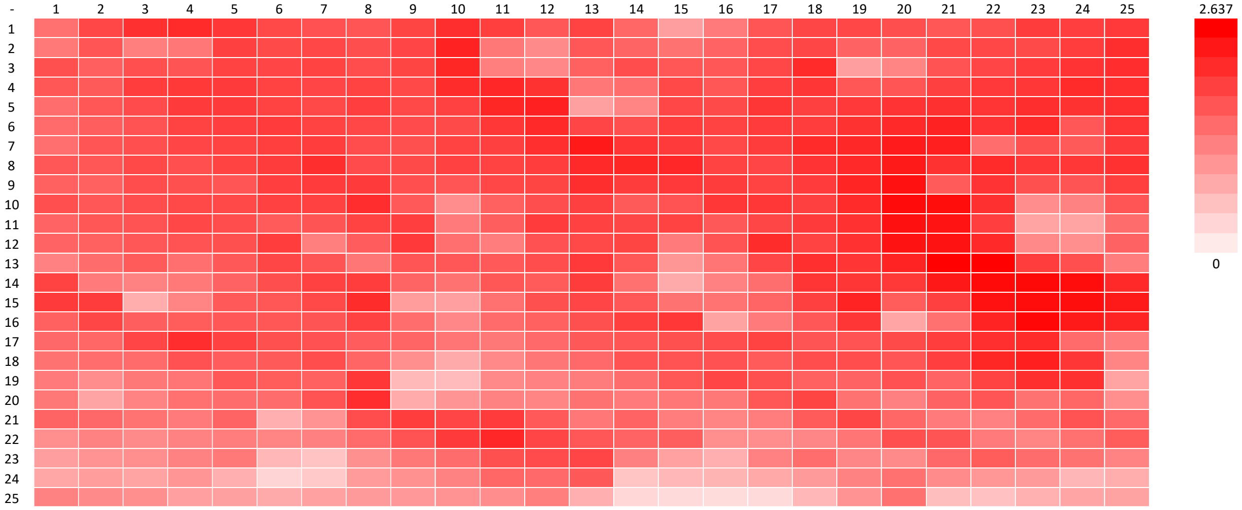

Fig. S34. UV-vis absorption 2D map of film of 1 from TCE (25 × 25 grid array area of 0.2 mm step size at 237 nm; top side).

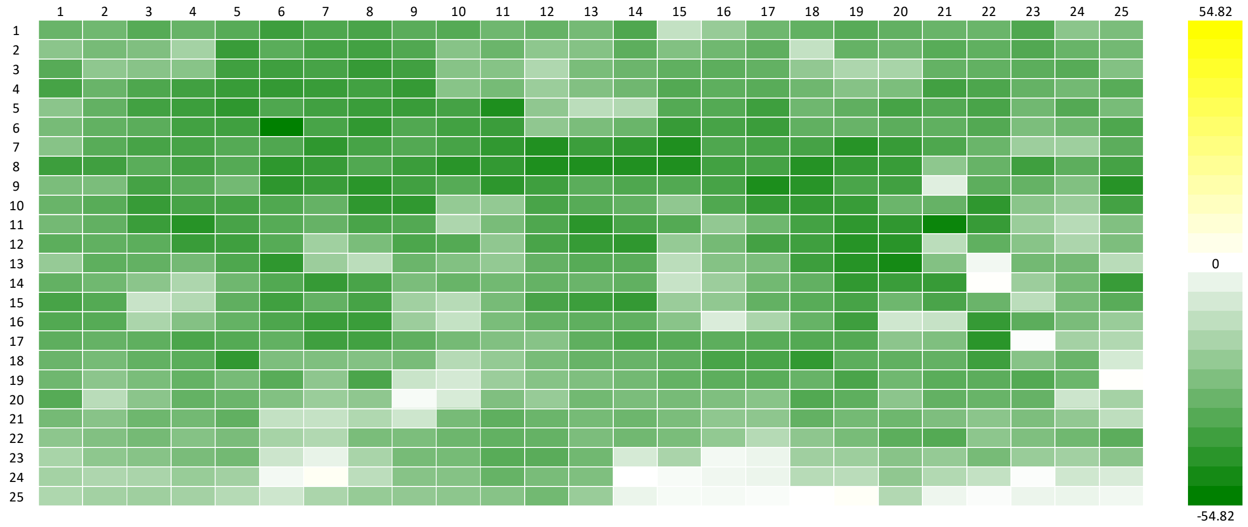

Fig. S35. ECD 2D map of film of 1 from TCE (25 × 25 grid array area of 0.2 mm step size at 237 nm; top side).

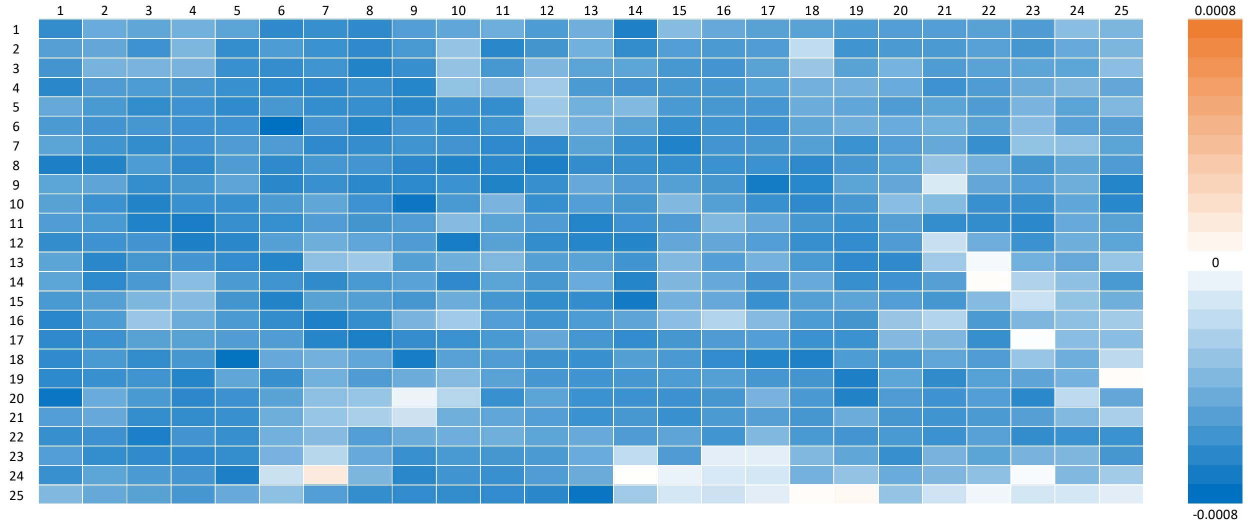

Fig. S36. Dissymmetry g-factor 2D map of film of 1 from TCE (25 × 25 grid array area of 0.2 mm step size at 237 nm; top side).

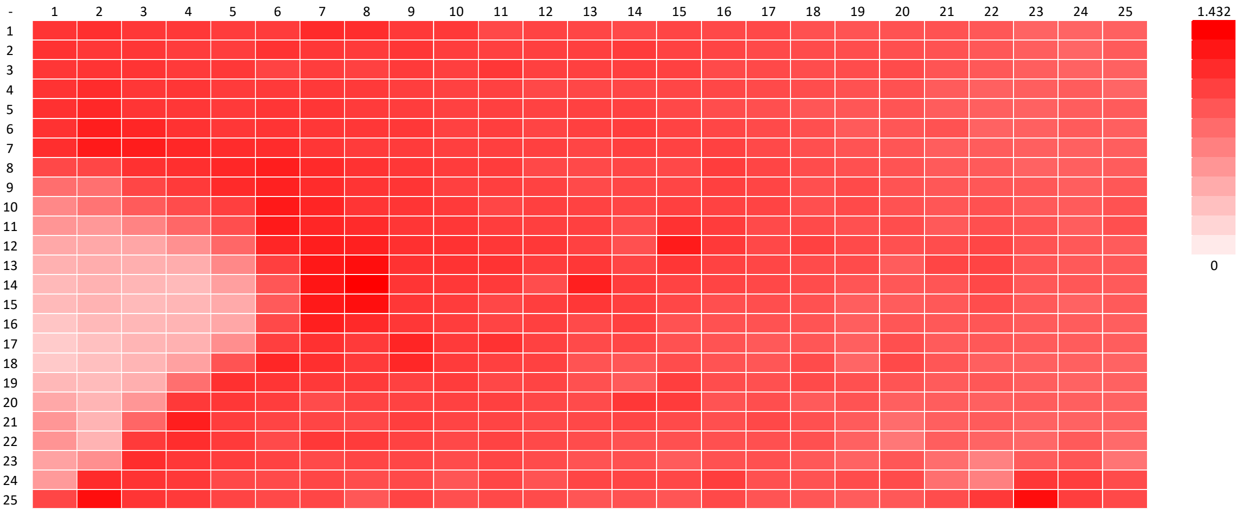

Fig. S37. UV-vis absorption 2D map of 2 of film from TCE (25 × 25 grid array area of 0.2 mm step size at 391 nm; top side).

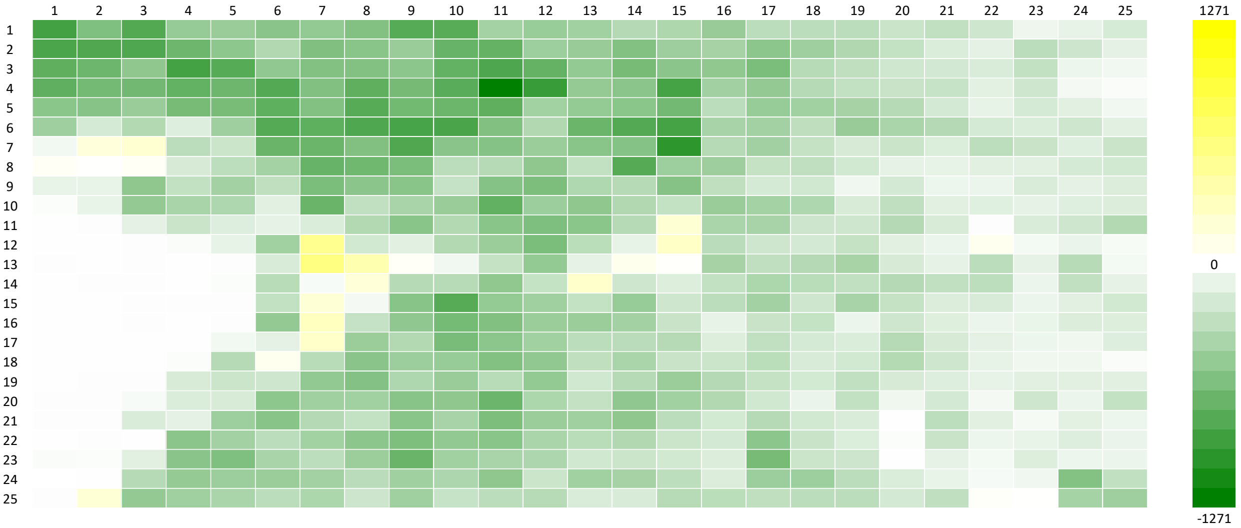

Fig. S38. ECD 2D map of 2 of film from TCE (25 × 25 grid array area of 0.2 mm step size at 391 nm; top side).

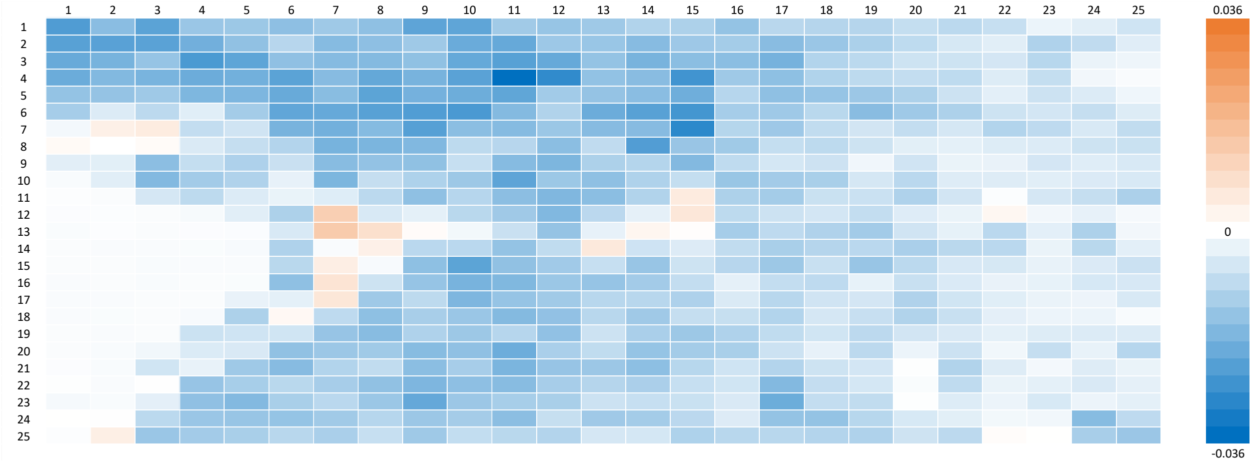

Fig. S39. Dissymmetry g-factor 2D map of 2 of film from TCE (25 × 25 grid array area of 0.2 mm step size at 391 nm; top side).

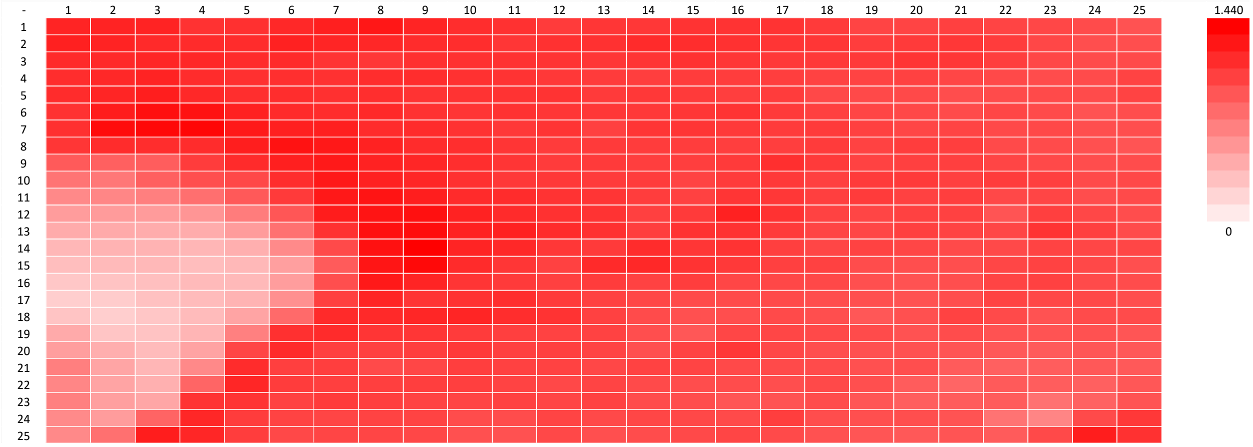

Fig. S40. UV-vis absorption 2D map of 2 of film from TCE (25 × 25 grid array area of 0.2 mm step size at 311 nm; top side).

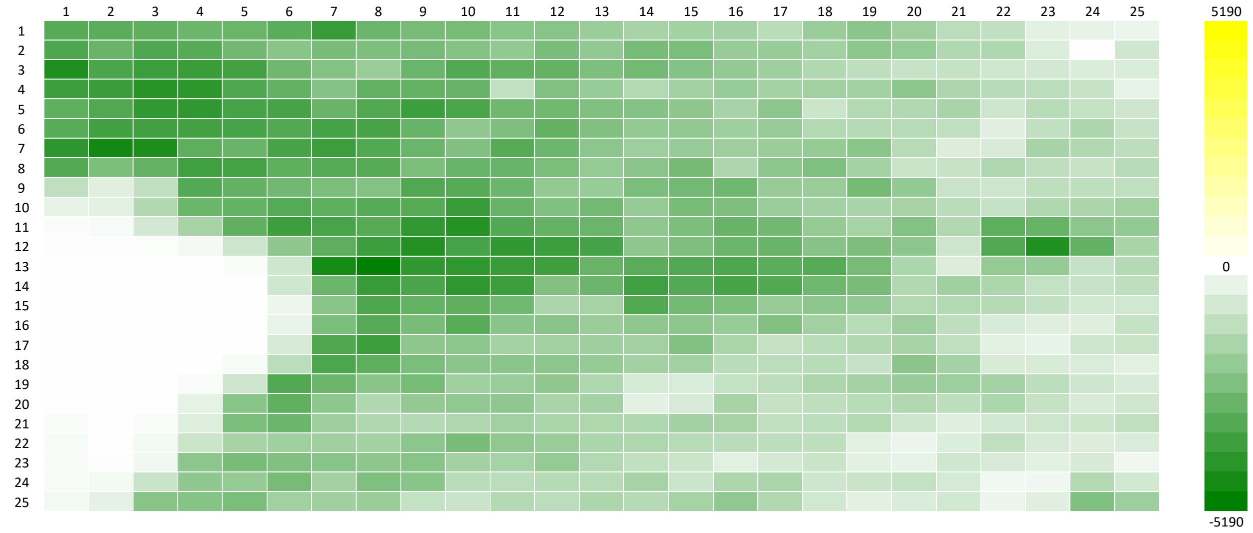

Fig. S41. ECD 2D map of 2 of film from TCE (25 × 25 grid array area of 0.2 mm step size at 311 nm; top side).

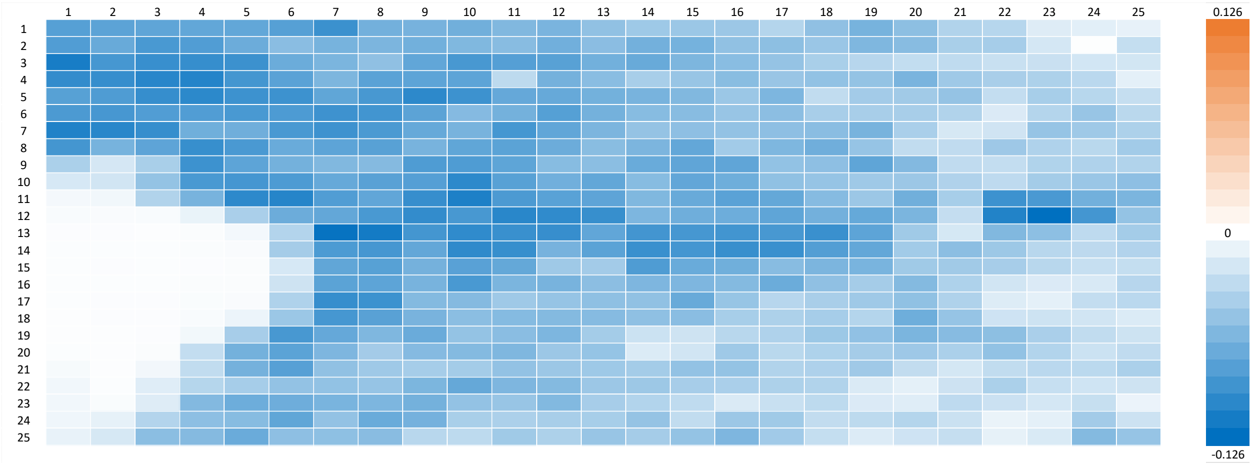

Fig. S42. Dissymmetry g-factor 2D map of 2 of film from TCE (25 × 25 grid array area of 0.2 mm step size at 311 nm; top side).

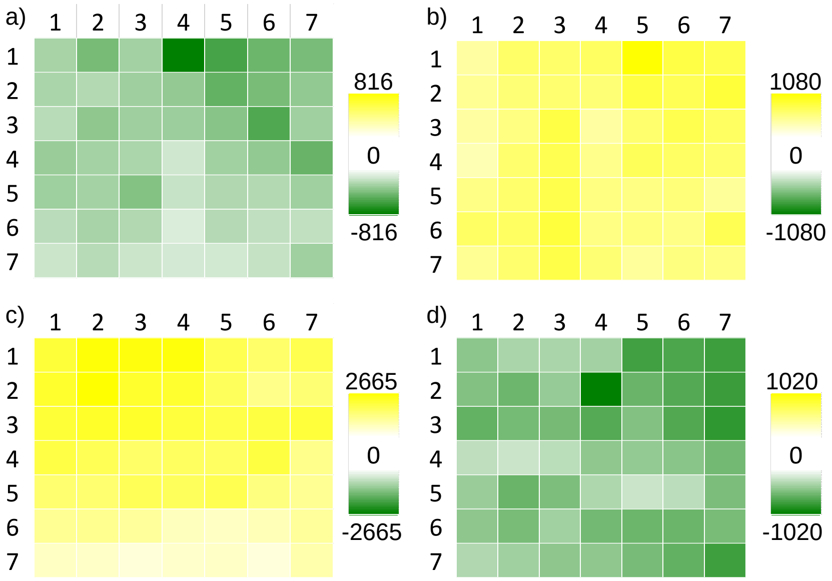

Fig. S43. ECD 2D CDi maps of film of 3 from TCE (7 × 7 grid array area of 0.2 mm step size) a) top side at 391 nm, b) bottom side at 391 nm, c) top side at 311 nm, d) bottom side at 311 nm.

4. 3D map of film of 1

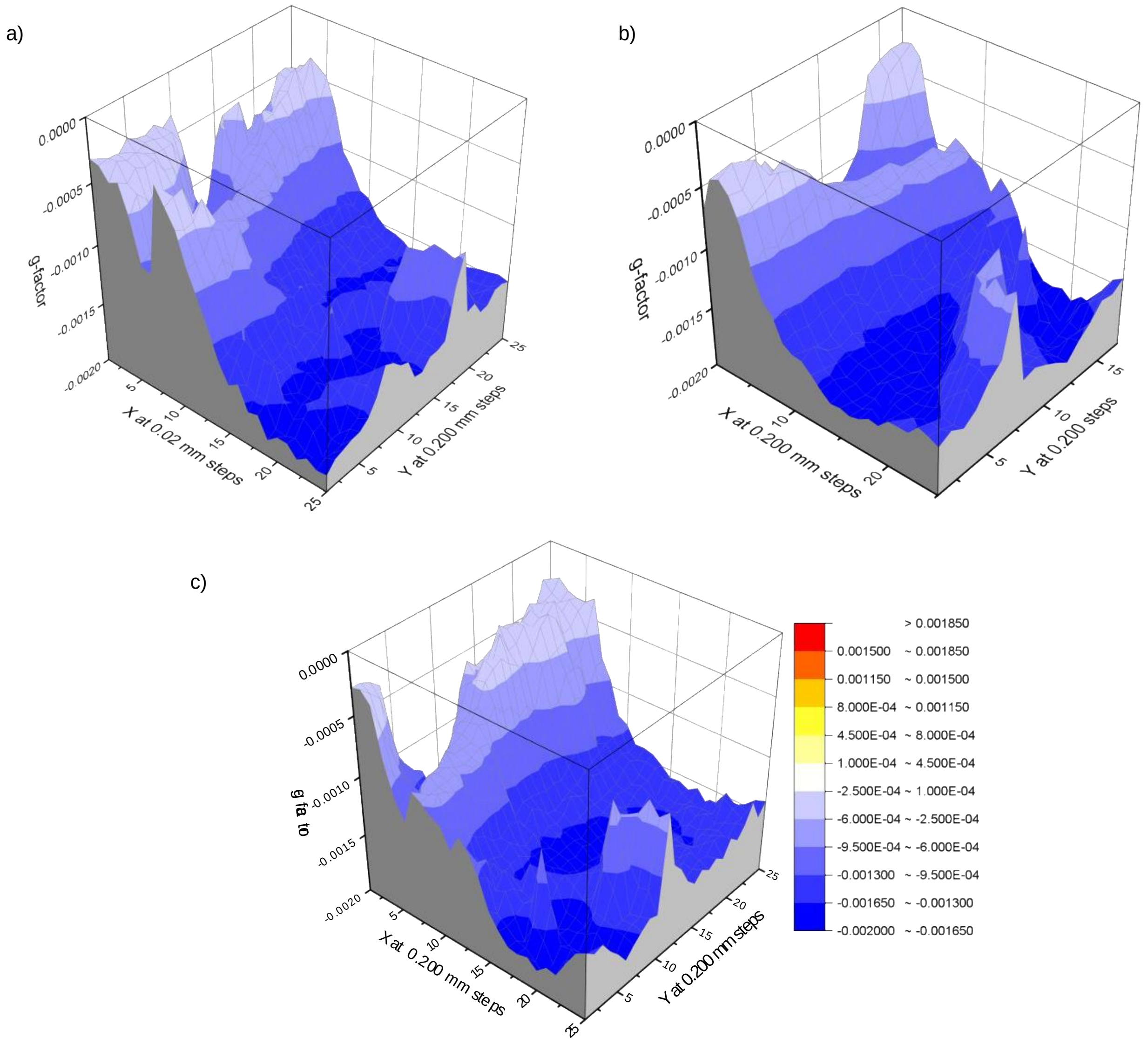

Fig. S44. 3D g-factor maps of film of 1 from DMF at 391 nm: a) top, b) bottom, c) bottom flipped along the y axis, indicating good degree of homogeneity and lack of LDLB contributions.

5. Thickness of film of 1

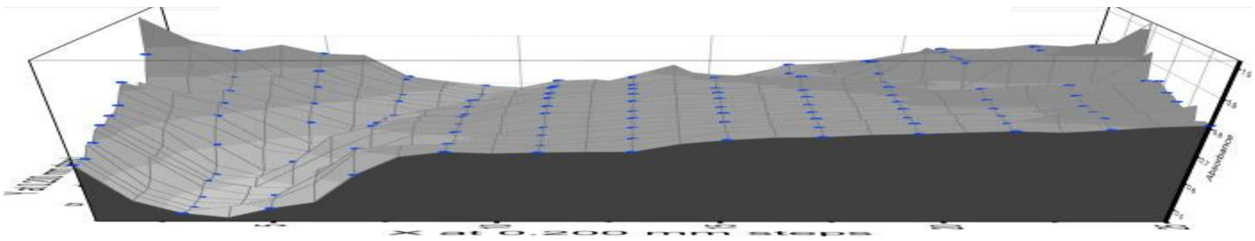

Fig. S45. Thickness-related measured by absorption of film of 1 from DMF; *x*- and *y*-axes are represented at 0.2 mm steps, while the *z*-axis is represented in Absorbance units.
